# Functional and Structural Alteration of Default Mode, Executive Control, and Salience Networks in Alcohol Use Disorder

**DOI:** 10.3389/fpsyt.2021.742228

**Published:** 2021-10-20

**Authors:** Ji-Woo Suk, Soonjo Hwang, Chaejoon Cheong

**Affiliations:** ^1^Department of Psychiatry, University of Nebraska Medical Center, Omaha, NE, United States; ^2^Bio-Chemical Analysis Team, Korean Basic Science Institute, Cheongju, South Korea

**Keywords:** alcohol use disorder, salience network, functional connectivity, gray matter volume, resting-state fMRI, voxel-based morphometry

## Abstract

Alcohol use disorder (AUD) has been related to aberrant functional connectivity (FC) in the salience network (SN), executive control network (ECN), and default mode network (DMN). However, there is a lack of comprehensive and simultaneous examination of these networks in patients with AUD and of their relation to potential anatomical changes. We aimed to comprehensively examine the alteration in FC in the three networks in AUD patients, and the correlation of the alteration with anatomical/structural changes (volume) in the neural areas implicated in these networks, by applying voxel-based morphometry (VBM) and region of interest-to-region of interest connectivity analysis simultaneously. In all, 22 patients with AUD and 22 healthy adults participated in the study and underwent T1 magnetic resonance imaging. Patients with AUD showed increased FCs within the DMN and SN networks, especially in terms of connectivity of the frontal areas and bilateral hippocampi. They also showed decreased FCs in the ECN. In addition, there was significant volume reduction in these areas (frontal areas and hippocampus). The increased FCs within the frontal areas or bilateral hippocampi showed a negative correlation with gray matter volume of these areas in AUD patients. Our findings add to the empirical evidence that the frontal lobe and hippocampi are critical areas that are vulnerable to functional and structural changes due to AUD.

## Introduction

Alcohol use disorder (AUD) is a chronic relapsing brain disease manifested by excessive alcohol consumption. Alcohol use disorder has a variety of negative social and health consequences, which are significant burdens on society (1, 2). As for the pathophysiology of AUD, numerous neuroimaging studies have been implemented to identify aberrations in brain structure and function related to AUD (3–5).

To date, most neuroimaging studies have focused on cognitive impairments in neural areas implicated in compulsivity, memory declining, and executive function impairment. These findings have suggested the frontal lobe hypothesis, wherein the prefrontal cortex, which plays a significant role in various cognitive functions, is predominantly vulnerable to the effects of alcohol (6, 7). However, the diffuse brain hypothesis states that damage caused by AUD can extend beyond the frontal lobe, extending to the areas such as the cerebellum, limbic system, and basal ganglia (8, 9). In this regard, recent resting-state functional magnetic resonance imaging (rs-fMRI) studies have provided additional perspectives by demonstrating system-level alterations in various regions of the brain as opposed to previous studies that focused on dysfunction in a specific region related to AUD (10).

In addition, emerging concepts in neuroimaging studies have suggested that pathophysiology of AUD involves the interaction of motivational, affective, and cognitive processes and multiple brain regions, rather than impairment in solely the cognitive process (5, 10). This is further supported by the clinical manifestation of AUD that includes a variety of symptoms such as disrupted reward anticipation, negative emotionality, dysfunctional cue reactivity, impulsivity, and compulsivity in addition to impaired executive function (5). Thus, there is a significant need for clinical and research work to comprehensively identify the alteration in neural networks relevant to motivational, affective, and cognitive processes to understand the neurophysiological mechanisms underlying the multiple symptoms of AUD.

The triple network model (11) was proposed to provide a common framework to understand the core dysfunctions in neurocognitive networks related to addiction and AUD in terms of the network approach (12). In this theoretical frame, three networks are implicated in the pathophysiology of addiction/AUD: (1) the executive control network (ECN), a fronto-parietal system composed of the dorsolateral prefrontal cortex (DLPFC) and interior parietal gyrus (IPG), implicated in manipulating information about the external environment (13); (2) the default mode network (DMN), comprising the ventromedial prefrontal cortex (vmPFC), and posterior cingulate cortex (PCC), implicated in monitoring self-referential mental processes (14); and (3) the salience network (SN), comprising the prefrontal cortex and anterior insula as key nodes, implicated in switching between the ECN and DMN by detecting, filtering, and integrating external stimuli and internal signals and allocating attentional resources between them (13, 15, 16).

Many studies on AUD that have used rs-fMRI have consistently identified abnormalities in the DMN (17–19), ECN (18–20), and SN (18, 19, 21). Prior studies on substance use disorders revealed the pattern of increased connectivity between midline core DMN (i.e., PCC) and medial temporal DMN (i.e., hippocampus and parahippocampal gyrus) (18, 22), and suggested that it might be involved in the conditioning of internal affective states with the experience of drug intake (23, 24). The studies on ECN of substance use reported the weaker connection in the DLPFC-parietal cortex and is an association with relapse to substance use (20, 25, 26). Increased functional connectivity (FC) of the insula in the SN, specifically the insula-anterior cingulate cortex (ACC), was exhibited in addiction disorders and implicated in compulsive wanting and motivation for addicted objects (19).

In particular, one of these studies suggested that the maladaptive decision making in individuals with AUD might be related not only to a deficiency in either the DMN or ECN but also to the difficulty in switching between those networks caused by impairment of the anterior insula, which is the key node in the SN (27). These findings indicate that the triple network plays a critical role in the pathophysiology of AUD. However, the majority of the previous studies focused on a single network and its relation to AUD. Thus, it is hard to determine whether altered FC in one network is the result of the damage to one of the brain regions in the network *per se* or the impact of dysfunctions/damage in other networks. In addition, the exact nature of the dysfunctions in FC of these networks in AUD is unclear and is attributed to reduced connectivity or increased connectivity (17–21). None of the studies has explored the association between anatomical aberration (volume changes) and FC dysfunction in these networks (17–21). These issues warrant the need for comprehensively and simultaneously examining the degree of FC in the key nodes of these networks and the interaction between them that may contribute to the neurobiology of AUD. To address these issues, we used rs-fMRI in combination with voxel-based morphometry (VBM) to examine the three networks implicated in the pathophysiology of AUD.

Recently, meta-analysis study on the structural findings of AUD using an effect-size based meta-analytical approach demonstrated significant GM reductions in the corticostriatal-limbic circuits including the key areas of the triple networks, such as DLPFC, hippocampus, bilateral insula, and ACC compared to healthy controls (9). Another study applying a different methodology (i.e., Anatomical Likelihood Estimation) also found gray matter reduction in the insula, ACC, and DLPFC, and demonstrated the association between volume reduction and functional impairment including cognition, emotion, and perception (28). This finding suggests that the gray matter reduction of the areas in the triple networks could be associated with cognitive and affective impairment observed in patients with AUD. However, to our knowledge, there is no study so far to directly identify the relationship between structural reduction and FC alteration in the triple networks of patients with AUD. Therefore, we aimed to identify volume changes by VBM in the three networks and provide further insight into the association between functional impairment and structural damage related to alcohol use in these neural areas (9, 29–31).

Based on previous findings, we hypothesized that AUD would lead to increased FC in the insula—ACC of the SN, in the PCC—hippocampus of the DMN, and decreased FC in the DLPFC—parietal cortex of the ECN (18–20). In addition, we expected individuals with AUD to exhibit gray matter reduction in the insula and ACC of the SN, in hippocampus of the DMN, and in the DLPFC of the ECN (7–9). We also expected the degree of structural change and functional dysfunction in the neural areas implicated in these networks to be significantly correlated.

## Materials and Methods

### Participants

Twenty-two participants with AUD and 22 healthy volunteers aged between 33 and 68 years participated in this study. The participants in the AUD group were recruited from the outpatient clinic or AA meeting in the local area. All participants in the AUD group were in the recovery or maintenance phase (at least five or more months after the detoxification phase). Healthy volunteers matched for age, education level, and smoking status, with no history of significant medical illness or psychiatric disorders, were included for comparison (Table 1).

**Table 1:** Demographic and clinical characteristics of the AUD and HC groups.

	**AUD** **(***N*** = 22)**	**HC** **(***N*** = 22)**	***t*** **or chi**
Age	49.818 (5.852)	50.174 (6.719)	0.18
Sex (male), *N* (%)	20 (90.9)	20 (90.9)	0
Years of education	11.909 (2.068)	12.565 (2.842)	0.888
AUDIT-K	35.591 (4.953)	12.217 (8.806)	10.026^***^
BDI	17.910 (8.646)	8.652 (8.912)	3.531^***^
Duration of illness	10.591 (5.105)	–	–
Number of hospital admission	3.591 (1.098)	–	–
History of neurologic symptoms^a^	12 (60)	–	–
(yes), N (%)			

*Values are means (standard deviation) unless indicated otherwise. AUD, alcohol use disorder; AUDIT, alcohol use disorder identification test; BDI, Beck depression inventory; HC, healthy controls*.

a *History of withdrawal convulsions or hallucinations*.

*** *p < 0.001 for group comparisons*.

All the participants were assessed using the Structured Clinical Interview for DSM-5, to determine that they met the criteria for alcohol dependence; the healthy participants did not meet any of the DSM-5 criteria for current axis I disorders. Participants with IQ scores below 80 measured by WAIS-IV (Wechsler Adult Intelligence Scale, Fourth Edition) (32), current symptoms of neurological abnormalities, history of psychotic symptoms, current use of any psychotropic medication, and conditions that would preclude MRI scans (i.e., claustrophobia or metal in the body) were excluded from the study.

All the participants provided written informed consent after receiving an explanation of the study aim and purpose. The research protocol was approved by the Institutional Review Board of the Korea Basic Science Institute (IRB approval ID: KBSI-IRB-2017-01). Data were generated at Korea Basic Science Institute. The data supporting the findings of the study are available from the first or corresponding author on request.

### Measurement Instruments

Demographic and clinical characteristics, including duration of illness and number of hospital admissions, were recorded. In addition, all the participants were assessed using the Alcohol Use Disorder Identification Test in Korea (AUDIT-K), Obsessive Compulsive Drinking Scale in Korea (OCDS-K), and Beck Depression Inventory (BDI) (Table 1). The AUDIT-K is a reliable and valid measurement for assessing AUD severity, including consumption, dependence, and alcohol-related problems in the Korean population (Cronbach's alpha = 0.92), it consists of 10 items, with more than 26 scores being alcohol-dependent (33, 34).

The severity of depressive symptoms was assessed using the BDI, which consists of 21 items evaluating emotional, cognitive, physiological, and behavioral symptoms. Its reliability was validated for the Korean population in a previous study (Cronbach's alpha = 0.91) (35, 36).

### Data Acquisition

All data were collected using a 3-T Philips Achieva MRI scanner (Philips Healthcare, Best, Netherlands). T1-weighted anatomical images were acquired with the following parameters: repetition time (TR) = 280 ms, echo time (TE) = 14 ms, flip angle = 60°, field of view (FOV) = 24 × 24 cm^2^, matrix = 256 × 256, and slice thickness = 4 mm.

For resting-state scanning, 303 images were acquired with a T2^*^-weighted gradient echo-planar imaging sequence for 10 min, 6 s (TR/TE = 2000/14 ms, flip angle = 80°, FOV= 24 × 24 cm^2^, matrix = 64 × 64, slice thickness = 4 mm without gap, 35 slices). All participants were instructed to keep their eyes closed, relax, and avoid falling asleep during the resting-state scanning.

### Selection of Regions of Interest

For FC and VMB analysis, the following ROI masks were defined: (1) the anterior SN [i.e., the left middle frontal gyrus, left insula, ACC, medial prefrontal cortex (mPFC), supplementary motor area (SMA), right middle frontal gyrus, right insula, left lobule VI, crus I, and left lobule VI, crus I]; (2) the posterior SN [i.e., the left middle frontal gyrus, left supramarginal gyrus (SMG), IPG, left precuneus, right midcingulate cortex, right superior parietal gyrus and precuneus, right SMG and IPG, left thalamus, right lobule VI, left posterior insula and putamen, right thalamus, left lobule VI, and right posterior insula]; (3) the dorsal DMN (i.e., the mPFC, ACC, and orbitofrontal cortex, left angular gyrus, right superior frontal gyrus, PCC and precuneus, right superior frontal gyrus, midcingulate cortex, right angular gyrus, thalamus, left hippocampus, and right hippocampus); (4) the ventral DMN (i.e., the left retrosplenial cortex and PCC, left middle frontal gyrus, left parahippocampal gyrus, left middle occipital gyrus, right retrosplenial cortex and PCC, precuneus, right superior frontal gyrus and middle frontal gyrus, right parahippocampal gyrus, right angular gyrus and middle occipital gyrus, left parahippocampal gyrus, right angular gyrus, and right lobule IX); (5) the left ECN (i.e., the left middle frontal gyrus and superior frontal gyrus, left inferior frontal gyrus and orbitofrontal gyrus, left superior parietal gyrus, IPG, precuneus, angular gyrus, left inferior temporal gyrus and middle temporal gyrus, right crus I, and left thalamus); and (6) the right ECN (i.e., the right middle frontal gyrus and superior frontal gyrus, right middle frontal gyrus, right IPG, SMG, and angular gyrus, right superior frontal gyrus, and crus I, II, and lobule VI, and caudate) as defined by the Stanford Atlas of Functional ROI (http://findlab.stanford.edu/functional_ROIs.html) (37).

### Functional Connectivity Analysis

The resting-state functional connectivity (rsFC) analysis was conducted using the CONN toolbox v.15 (http://www.nitrc.org/projects/conn). Preprocessing was performed using the standard CONN pipeline, which included realignment with three-dimensional rigid body registration with six degrees of freedom, coregistration to each participant's anatomical scan, slice-time correction, structural segmentation, normalization to the standard brain template with the Montreal Neurological Institute space, and spatial smoothing using an 8-mm full width at half maximum isotropic Gaussian kernel. White matter (WM) and cerebrospinal fluid signals were regressed from the data using CompCor (38). After detrending to remove the systematic drift or trend, a band-pass filter (0.01–0.08 Hz) was applied to reduce the effect of low-frequency drift and high-frequency physiological signal or noise.

After the aforementioned preprocessing steps, rsFC analysis with ROI-to-ROI analysis with the same ROIs as the VBM analysis was performed using the CONN toolbox, followed by *post-hoc* analyses using the Statistical Package for the Social Sciences (SPSS, version 21.0). ROI-to-ROI analysis at an individual subject level was conducted by calculating the BOLD signal temporal correlations for all 198 pair-wise ROI combinations, see Supplementary Material Section 1.

For statistical analyses within the groups, each participant's functional brain connectivity map was generated with a threshold at the significance level of the whole-brain cluster, with a corrected alpha level of 0.05. For between-group comparisons, analysis of covariance (ANCOVA) was executed to compare *z*-value maps between participants with AUD and healthy controls after controlling for covariates such as age, sex, years of education, onset age of alcohol use, and BDI score. The thresholds for voxel-level height and cluster-level extent were set with an FDR-corrected *P* < 0.05. Seed-level correction was used to apply the FDR separately for each seed ROI by implementing both a voxel-level height threshold and a cluster-level extent threshold.

### Voxel-Based Morphometry Analysis

Voxel-based morphometry analysis was conducted using the Computational Anatomy Toolbox (CAT12; http://dbm.neuro.uni-jena.de/cat/), an extension toolkit of the Statistical Parametric Mapping software package (SPM12, Institute of Neurology, London, UK) running in MATLAB (R2019a; MathWorks, Natick, Massachusetts, USA).

All anatomical images were processed in the following steps: (1) visual examination for structural abnormalities and artifacts caused by head motion or dental instruments; (2) bias correction to remove MRI inhomogeneities; (3) segmentation into GM, WM, and cerebrospinal fluid (39); (4) registration to the standard Montreal Neurological Institute space included in a linear affine transformation and a non-linear deformation with diffeomorphic anatomical registration through exponentiated lie algebra normalization (40); (5) homogeneity check using covariance between normalized and segmented images; and (6) spatial smoothing with an 8-mm full width at half maximum Gaussian kernel.

After preprocessing, the GM volumes of the ROIs were compared between the AUD and control groups. Analysis of covariance was used to identify brain regions within the masks of salience, executive control, or DMNs that had GMV differences [*P* < 0.01, false discovery rate (FDR)-corrected] between the groups. To exclude the effects of nuisance variables on the structural alteration, sex, age, depression, onset age of alcohol use, total intracranial volume (TIV), and IQ were added as additional covariates.

### Correlation Analysis Between FC and Gray Matter Volume

To examine whether the alterations of FCs are associated with anatomical aberrations, the bivariate Pearson correlation analyses were conducted using the FC-value and gray matter density in each ROI among individuals with AUD using SPSS version 26 (IBM Corp., Armonk, NY USA).

## Results

### Participant Characteristics

Participants with AUD and healthy controls did not differ significantly in age (*t* = 0.18, *p* > 0.05), sex (*x*^2^ = 0, *p* > 0.05), or years of education (*t* = 0.888, *p* > 0.05). As expected, individuals with AUD scored higher self-reported BDI (*t* = 3.531, *P* < 0.001), AUDIT-K (*t* = 10.026, *P* < 0.001), and OCDS-K (*t* = 6.861, *P* < 0.001) than the healthy controls (Table 1).

### Functional Connectivity Analysis

#### Connectivity in Salient Network

Individuals with AUD had significantly increased FC of the left insula—the ACC, SMA, and mPFC (*t* = 2.67, *P* < 0.05) in the anterior SN compared to healthy adults. In the posterior SN, decreased connectivity of the right thalamus—the left SMG and IPG was observed in the AUD group (*t* = −3.66, *P* < 0.05) (Table 2; Supplementary Figures 1, 2).

**Table 2:** Difference in functional connectivity in each network between the groups.

**Network**	**Regions**		**AUD** **(***N*** = 22)**	**HC** **(***N*** = 22)**	***t***
ASN	L. INS	*ACC, SMA, mPFC*	0.77	0.64	2.67^*^
PSN	R. Th	R. SMG, IPG	0.00	0.13	−3.66^**^
DDMN	*R. Hip*	R. AG	0.23	0.10	3.28^*^
	*R. Hip*	*L. Hip*	0.70	0.57	2.91^*^
	*L. Hip*	R. AG	0.21	0.07	2.92^*^
VDMN	R. SFG, MFG	*R. PHG*	0.29	0.13	3.34^*^
	R. AG, MOG	L. MFG	0.24	0.45	−3.40^*^
	R. AG, MOG	*R. PHG*	0.38	0.23	3.09^*^
RECN	R. IPG, SMG, AG	R. MFG, SFG	0.94	1.16	−2.82^*^

*Values are correlation coefficients unless indicated otherwise. The italicized regions showed the reductions of gray matter volume among AUD group. ACC, anterior cingulate cortex; AG, angular gyrus; ASN, anterior salience network; AUD, alcohol use disorder group; DDMN, dorsal default mode network; HC, healthy control group; INS, insula; IPG, inferior parietal gyrus; Hip, hippocampus; L, left; MFG, middle frontal gyrus; mPFC, medial prefrontal cortex; MOG, middle occipital gyrus; PHG, parahippocampal gyrus; PI, posterior insula; PSN, posterior salience network; R, right; RECN, right executive control network; SFG, superior frontal gyrus; SMA, supplementary motor area; SMG, supramarginal gyrus; Th, thalamus; VDMN, ventral default mode network*.

* *p < 0.05 and*

** *p < 0.01 for group comparisons*.

#### Connectivity in Default Mode Network

Compared to the healthy group, the AUD group showed increased connectivity in (1) the right hippocampus—right angular cortex (*t* = 3.28, *P* < 0.05); (2) right hippocampus—left hippocampus (*t* = 2.91, *P* < 0.05); and (3) left hippocampus—right angular cortex (*t* = 2.92, *P* < 0.05) of the dorsal DMN (Table 2; Supplementary Figure 3). In the ventral DMN, the right angular gyrus and middle occipital gyrus—left middle frontal gyrus connection (*t* = −3.40, *P* < 0.05) had increased and (1) right superior frontal gyrus and middle frontal gyrus—right parahippocampal gyrus (*t* = 3.34, *P* < 0.05); (2) right parahippocampal gyrus—right angular gyrus and middle occipital gyrus (*t* = 3.09, *P* < 0.05) had decreased in the AUD group (Table 2; Supplementary Figure 4).

#### Connectivity in Executive Control Network

In addition, in the right ECN, the FCs of the right IPG, SMG, and angular gyrus—right middle frontal gyrus and superior frontal gyrus (*t* = 2.82, *P* < 0.05) were decreased in the AUD group compared to healthy adults. There was no significant difference in the FC in the left ECN between the groups (Table 2). Supplementary Tables 7–12 show the correlation coefficients between the ROIs in each network and each group, and Supplementary Tables 13–18 represent group differences in the FC in each network.

### VBM Analysis

The AUD group had lower gray matter volume in the three networks than the healthy control group: (1) the ACC, mPFC, SMA (*t* = 2.180, *P* < 0.05) in the anterior SN; (2) the left thalamus (*t* = 2.025, *P* < 0.05), left lobule VI (*t* = 2.104, *P* < 0.05), and right posterior insula (*t* = 2.946, *P* < 0.05) in the posterior SN; (3) the mPFC, ACC, orbitofrontal cortex (*t* = 2.117, *P* < 0.05), left hippocampus (*t* = 2.896, *P* < 0.05), and right hippocampus (*t* = 2.828, *P* < 0.05) in the dorsal DMN; (4) the left parahippocampal gyrus (*t* = 2.943, *P* < 0.05), right parahippocampal gyrus (*t* = 2.633, *P* < 0.05), and right lobule IX (*t* = 2.100, *P* < 0.05) in the ventral DMN; (5) the left middle frontal gyrus, superior frontal gyrus (*t* = 2.281, *P* < 0.05), and left inferior frontal gyrus and orbitofrontal gyrus (*t* = 2.811, *P* < 0.05) in the left ECN; and (6) the left crus I, II, lobule VI (*t* = 2.491, *P* < 0.05) in the right ECN (*P* < 0.05, FDR-corrected; Table 3). The group differences in GM volume in all ROIs of each network are listed in the Supplementary Tables 1–6.

**Table 3:** Difference in gray matter volume in each network between the groups.

**Network**	**Region**	**Side**	**BA**	**AUD** **(***N*** = 22)**	**HC** **(***N*** = 22)**	***t***
ASN	ACC, mPFC, SMA	B	6, 8, 24, 32	10.064 (1.247)	10.818 (1.003)	2.18^*^
PSN	Th	L		0.340 (0.070)	0.378 (0.053)	2.03^*^
	LVI	L		0.029 (0.009)	0.034 (0.010)	2.10^*^
	PI	R	48	0.609 (0.090)	0.676 (0.056)	2.95^**^
DDMN	mPFC, ACC, OFC	B	9, 10, 11, 24, 32	18.880 (2.036)	19.983 (1.565)	2.12^*^
	Hip	L	20, 30, 36	1.421 (0.091)	1.516 (0.119)	2.90^**^
	Hip	R	20, 30, 36	0.479 (0.037)	0.525 (0.045)	2.83^**^
VDMN	PHG	L	20, 37	0.695 (0.076)	0.768 (0.086)	2.94^**^
	PHG	R	30, 37	0.399 (0.047)	0.437 (0.047)	2.63^*^
	LIX	R		0.296 (0.048)	0.328 (0.048)	2.10^*^
LECN	MFG, SFG	L	8, 9	1.566 (0.230)	1.747 (0.283)	2.28^*^
	IFG, OFG	L	10, 45, 47	1.285 (0.174)	1.434 (0.172)	2.81^**^
RECN	CI, CII, LVI	L		8.849 (0.935)	9.605 (1.106)	2.40^*^

*Values are means (standard deviation) unless indicated otherwise. ACC, anterior cingulate cortex; ASN, anterior salience network; AUD, alcohol use disorder group; CI, Crus I; CII, Crus II; DDMN, dorsal default mode network; HC, healthy control group; Hip, hippocampus; L, left; LIX, lobule IX; LVI, lobule VI; MFG, middle frontal gyrus; mPFC, medial prefrontal cortex; LECN, left executive control network; OFG, orbitofrontal gyrus; PC, precuneus; PHG, parahippocampal gyrus; PI, posterior insula; PSN, posterior salience network; R, right; RECN, right executive control network; SFG, superior frontal gyrus; SMA, supplementary motor area; Th, thalamus; VDMN, ventral default mode network*.

* *p < 0.05 and*

** *p < 0.01 for group comparisons*.

### Correlation Analysis Between FC and Gray Matter Volume

**Figure 1:**
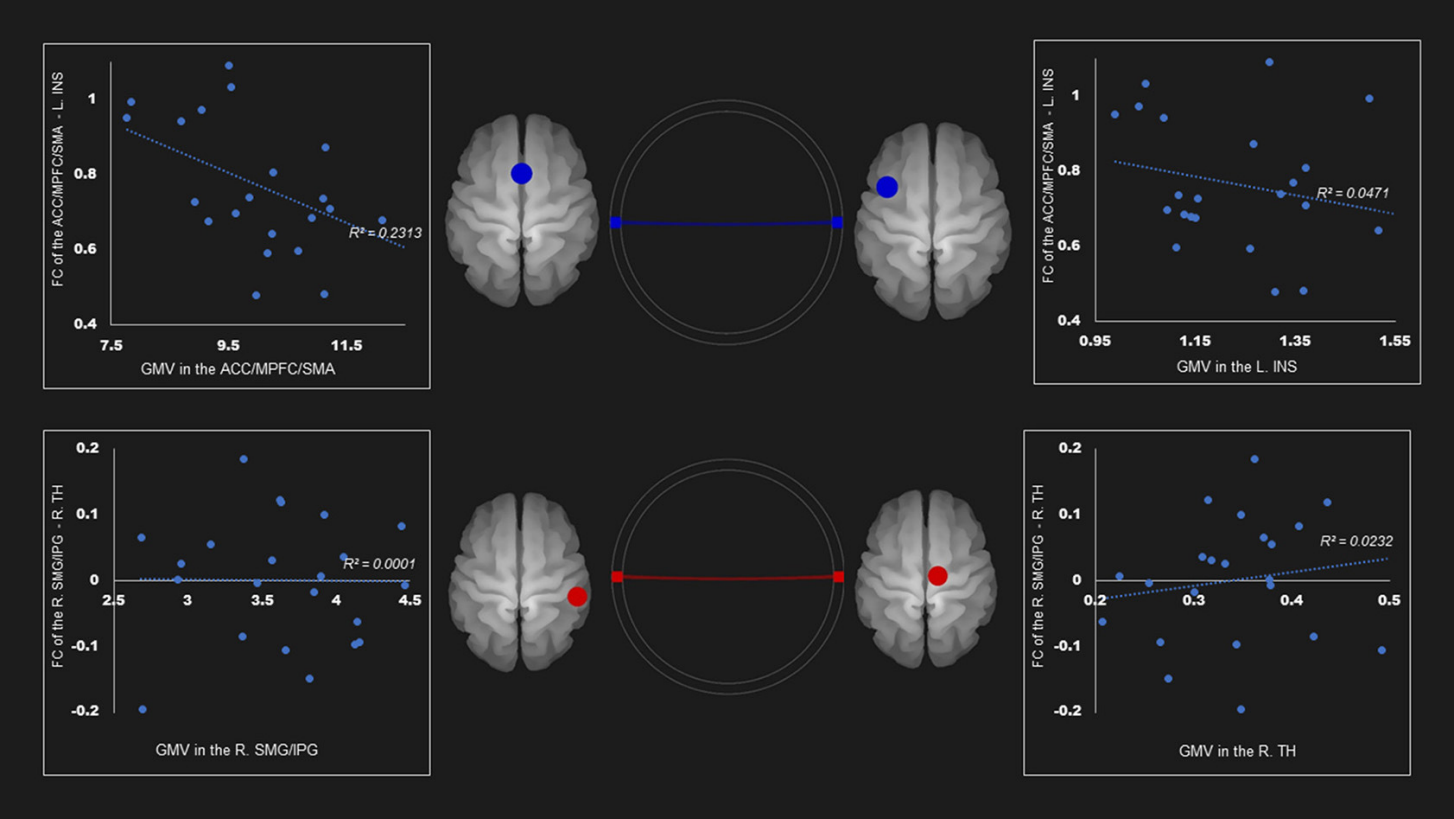
Correlation between FC and gray matter volume in the salience network among AUD group. Gray matter volume of anterior cingulate gyrus/medial prefrontal cortex/supplementary motor area was negatively associated with the FC of the left insula—anterior cingulate gyrus/medial prefrontal cortex/supplementary motor area *(r* = −0.48). Red and blue lines represent the positive and negative functional connectivity between ROIs, respectively. FC, functional connection; ACC, anterior cingulate cortex; AUD, alcohol use disorder group; INS, insula; L, left; MPFC, medial prefrontal cortex; R, right; SFG, superior frontal gyrus; TH, thalamus.

Table 4 and Supplementary Figures 1–4 show the correlations coefficients for the AUD group between gray matter volume and the FC in the ROIs; there were significant differences in both gray matter volume and FC between the groups. There was a negative correlation between the FC of left insula—ACC, SMA, and mPFC and the gray matter density of the ACC, SMA, and mPFC in the SN (*r* = −0.48, *P* < 0.05) (Table 4; Figure 1).

**Table 4:** The relationship between gray matter volume and functional connectivity in each network in the AUD group.

**Network**	**ROI I**	**ROI II**	**Correlation between FC and GMV of ROI I**	**Correlation between FC and GMV of ROI II**
ASN	L. INS	*ACC, SMA, mPFC*	−0.22	−0.48^*^
PSN	R. Th	R. SMG, IPG	0.15	−0.01
DDMN	*R. Hip*	R. AG	–0.55^**^	−0.20
	*R. Hip*	*L. Hip*	–0.45^*^	–*0.35*
	*L. Hip*	R. AG	–0.42^*^	0.02
VDMN	R. SFG, MFG	*R. PHG*	−0.02	–0.60^**^
	R. AG, MOG	L. MFG	0.05	−0.19
	R. AG, MOG	*R. PHG*	−0.05	–*0.39*
RECN	R. IPG, SMG, AG	R. MFG, SFG	0.00	0.14

*Values are correlation coefficient unless other indicated. ACC, anterior cingulate cortex; AG, angular gyrus; ASN, anterior salience network; AUD, alcohol use disorder group; Cau, caudate; CI, crus I; CII, crus II; DDMN, dorsal default mode network; HC, healthy control group; Hip, hippocampus; INS, insula; IPG, inferior parietal gyrus; L, left; LIX, lobule IX; LVI, lobule VI; MCC, midcingulate cortex; MFG, middle frontal gyrus; mPFC, medial prefrontal cortex; MOG, middle occipital gyrus; PC, precuneus; PCC, posterior cingulate cortex; PHG, parahippocampal gyrus; PSN, posterior salience network; R, right; RC, restrosplenial cortex; RECN, right executive control network; SFG, superior frontal gyrus; SMA, supplementary motor area; SMG, supramarginal gyrus; SPG, superior parietal gyrus; Th, thalamus; VDMN, ventral default mode network. The italicized regions showed the reductions of gray matter volume among AUD group*.

* *p < 0.05 and*

** *p < 0.01*.

**Figure 2:**
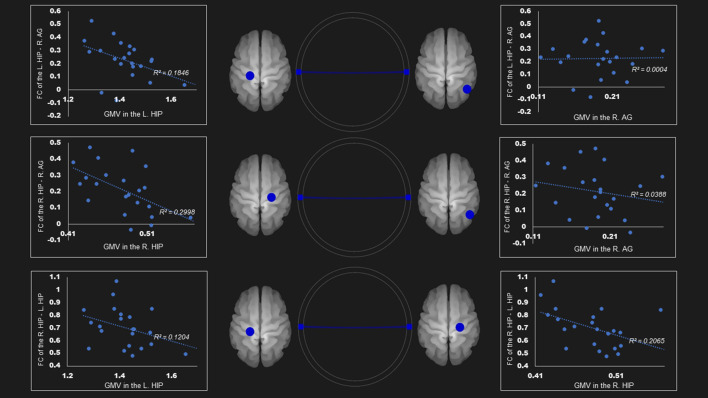
Correlation between FC and gray matter volume in the dorsal default mode network in the AUD group. Gray matter volume of right hippocampus was negatively associated with the FC of (1) the right hippocampus—right angular gyrus (*r* = −0.55); (2) the right hippocampus—the left hippocampus (*r* = −0.45). Left hippocampus volume was also negatively linked to the FC of (1) the left hippocampus—right angular gyrus (*r* = −0.43); (2) the right hippocampus—the left hippocampus (*r* = −0.35). Blue lines represent the negative functional connectivity between ROIs, respectively. FC, functional connection; AG, angular gyrus; AUD, alcohol use disorder group; Hip, hippocampus; L, left; R, right.

The gray matter volume of right hippocampus was negatively associated with the FC between (1) right hippocampus—right angular cortex (*r*= −0.55, *P* < 0.05); and (2) right hippocampus—left hippocampus (*r* = −0.45, *P* < 0.05) in the dorsal default network. The left hippocampus size was negatively linked to the FC between the left hippocampus—right angular cortex (*r* = −0.42, *P* < 0.05) (Table 4; Figure 2).

**Figure 3:**
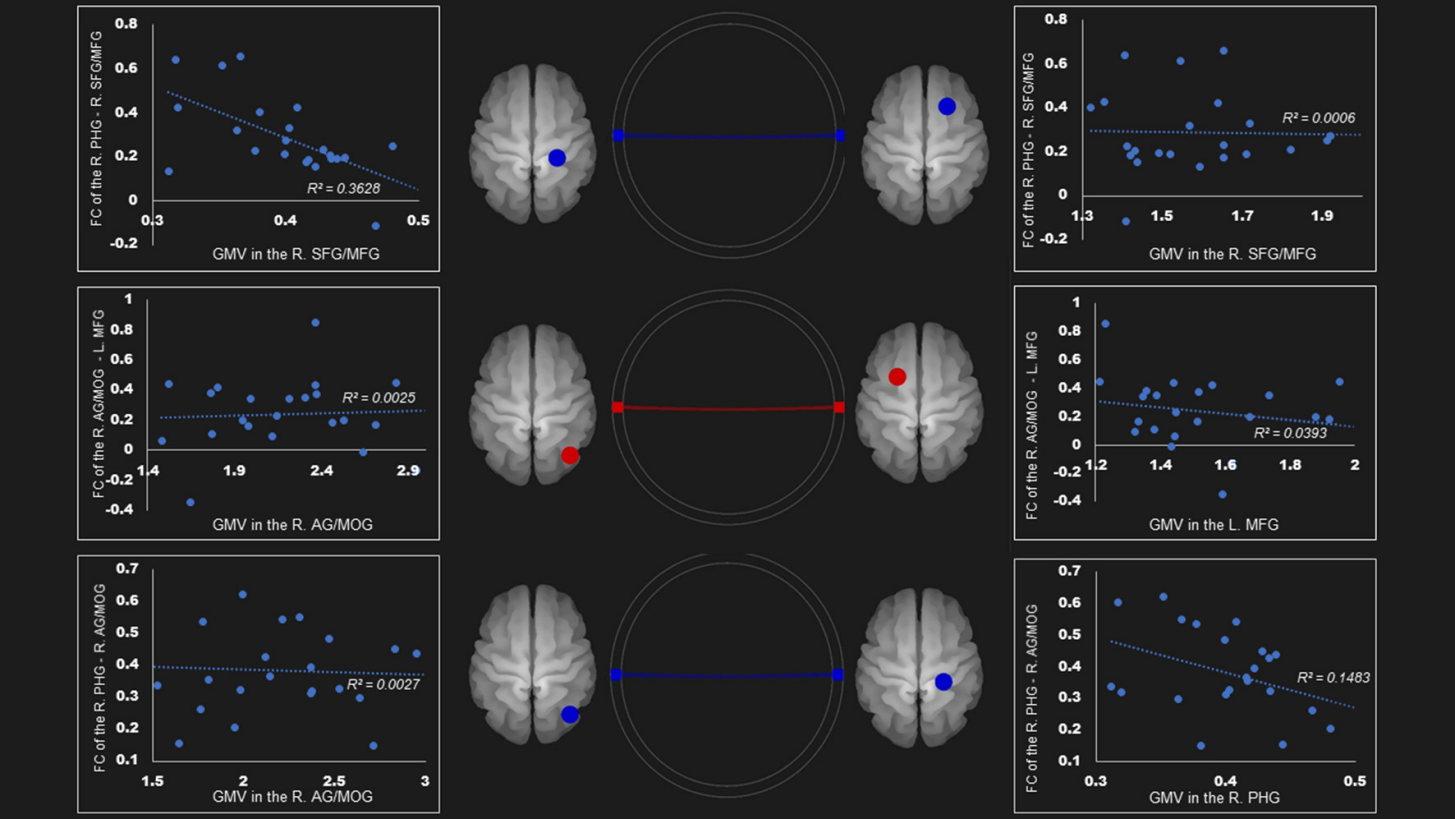
Correlation between FC and gray matter volume in the ventral default mode network in the AUD group. Gray matter volume of right parahippocampal gyrus was negatively associated with the FC of the (1) right parahippocampal gyrus—right superior frontal gyrus/middle frontal gyrus (*r* = −0.60); (2) right parahippocampal gyrus—right angular gyrus/middle occipital gyrus (*r* = −0.39). Red and blue lines represent the positive and negative FC between ROIs, respectively. FC, functional connection; AG, angular gyrus; AUD, alcohol use disorder group; L, left; MFG, middle frontal gyrus; MOG, middle occipital gyrus; PHG, parahippocampal gyrus; R, right; SFG, superior frontal gyrus.

In the ventral DMN, the gray matter density of right parahippocampal gyrus was negatively linked to the FC of right superior frontal gyrus and middle frontal gyrus—right parahippocampal gyrus (*r* = −0.60, *P* < 0.01) and the right angular gyrus and middle occipital gyrus—right parahippocampal gyrus (*r* = −0.39, *p* = 0.0596) (Figure 3).

**Figure 4:**
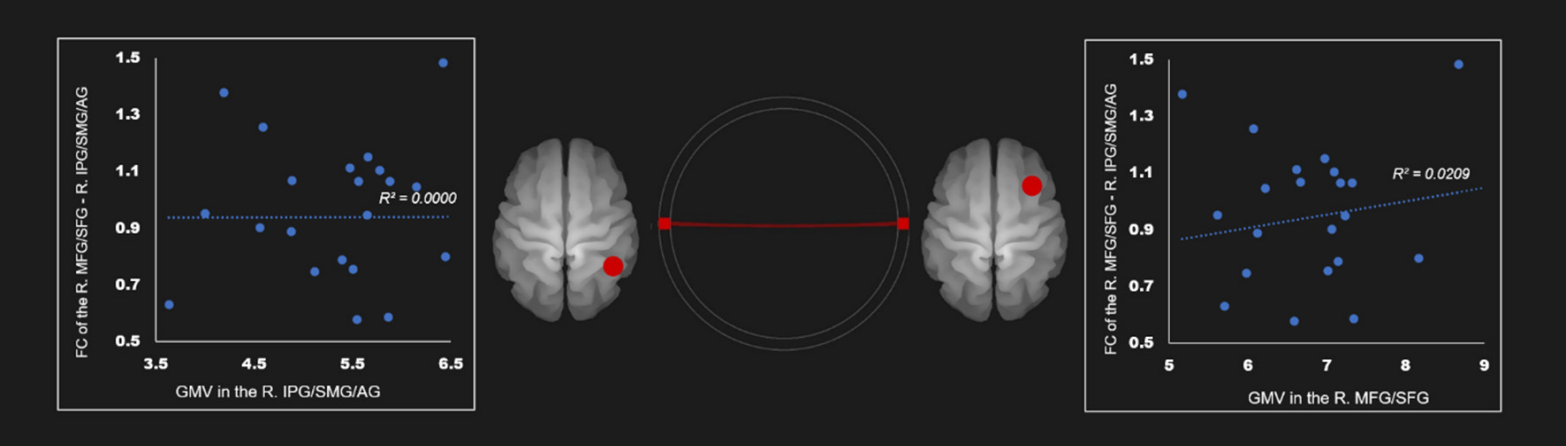
Correlation between FC and gray matter volume in the right executive control network in the AUD group. Red line represents the positive functional connectivity between ROIs, respectively. FC, functional connection; AG, angular gyrus; AUD, alcohol use disorder group; IPG, inferior parietal gyrus; MFG, middle frontal gyrus; R, right; SFG, superior frontal gyrus; SMG, supramarginal gyrus.

There was no correlation between the gray mater density and the FC of the right IPG, SMG, and angular gyrus—right middle frontal gyrus and superior frontal gyrus (Table 4; Figure 4). Supplementary Tables 19–24 show correlation coefficients for the association between gray matter volume and FC of all ROIs in each network in the AUD group.

## Discussion

In this study, we aimed to identify the alteration of FC in the SN, DMN, and ECN in AUD patients, and the correlation between impaired FC and structural changes (volume) in the neural areas implicated in these networks by applying VBM and ROI-to-ROI connectivity analysis simultaneously.

We have three main findings. First, patients with AUD showed increased FCs in the anterior SN, dorsal DMN, and ventral DMN compared to healthy adults. Patients with AUD also showed decreased FCs in the posterior SN, ventral DMN, and right ECN. Second, patients with AUD showed decreased gray matter volume in neural areas implicated in all the triple networks. Lastly, there was a negative correlation between the gray matter volume and FC in anterior SN, dorsal DMN, and ventral DMN.

As we predicted, the AUD group showed increased connections in the anterior SN, especially the FC between the left insula and ACC, SMA, and mPFC. The insula and ACC are key nodes in the SN, which separates the most relevant internal and extra-personal stimuli for guiding goal-directed behavior. In particular, the FCs between these regions facilitate rapid access to the motor system (13, 16, 19). According to the neuro-circuitry model of addiction (12, 41, 42), enhanced interactions between the insula and ACC may be related to elevated salience for addictive substance and related cues at the expense of other activities. As a result, the disrupted ACC–insula circuits may render individuals with AUD vulnerable to engaging with alcohol consumption.

Participants with AUD showed increased connectivity in the DMN, especially areas connected with the bilateral hippocampi. Previous studies on substance use disorders have demonstrated the critical role of the hippocampus in substance-related neuroplasticity and relapse (43–45). A previous study suggests that the hippocampus links with multiple cortico-striatal regions to impact memory and decision making (46). The hippocampus interacts with other striatal-limbic regions and plays an important role in drug-related contextual memories such as acquisition, consolidation, and retrieval of learning of addiction-related cues, which underlie the reinstatement of drug-seeking behaviors (47, 48). In addition, integrated inputs from the hippocampus, ACC, IPG, SMG, and prefrontal cortex generate motivational signals that modulate drug-related attentional bias and repeated drug self-administration (45, 49, 50). Thus, increased FC within the hippocampus might be associated with experience-dependent changes related to strong memories for alcohol-related cues or responses subsequent to alcohol consumption, rendering individuals vulnerable to frequent or strong cravings for alcohol.

The AUD group also demonstrated decreased FC in the ECN (especially between the right IPG, SMG, and angular gyrus and right middle frontal gyrus, superior frontal gyrus). Alteration in fronto-parietal connectivity is the most commonly observed finding in substance use disorders (51–53). Fronto-parietal connectivity mediates cognitive control functions, such as inhibitory control, planning, and complex decision making (54, 55). Combined together, these results indicate that the altered FC between the frontal area and hippocampus, as the key node, may be associated with excessive attentional bias to drug-related cues, preferential assignment to drug-seeking behavior, and repeated drug administration caused by loss of control.

The VBM result demonstrated structural impairment in all three networks. However, structural damage was mainly present in the frontal lobe, including the mPFC, SMA, orbitofrontal cortex, and left superior/middle/inferior frontal cortex involved in the anterior SN and right ECN. Reductions in hippocampal volume including bilateral hippocampus and parahippocampal gyrus were reported in the AUD group, even though none of the participants had a history of alcoholic Korsakoff syndrome. Gray matter density in the right posterior insula, thalamus, and ACC had also decreased. These results support the diffuse brain hypothesis suggesting diffuse damage in the cerebral cortex caused by the neurotoxic effects of alcohol (7, 10). The frontal lobe and hippocampus are rich in glucocorticoid receptors and considered particularly susceptible to the neuro-toxic effect of alcohol (56, 57), supporting the vulnerability of these regions (58, 59).

Moreover, there was significant level of negative correlation between gray matter volume reduction and changes in the FC in the AUD group especially in the ACC/SMA/mPFC, hippocampus, and hippocampal gyrus (Table 4). It is noteworthy that all the negative correlations between gray matter volume and FC were found only in the ROIs showing loss of gray matter volume (Supplementary Tables 19–24). Jansen et al. (60) suggested that increased FC in areas showing structural impairment is related to compensatory mechanisms for disruption of functional networks. In other words, it utilizes additional activation in FC of the implicated neural areas damaged by alcohol consumption evidenced by volume reduction. Previous studies have reported the effect of long-term consumption of alcohol on GM and WM. In these studies, when alcohol was consumed for a long time, volume reduction was observed in the prefrontal lobe, insular lobe, and anterior cortex, and hyperactivation was observed in these regions (61–64). We found that FC acts in the compensatory mechanism for volume reduction in the core areas of SN and DMN.

Furthermore, the increased FC due to the compensatory mechanism seems to cause a decline in other FC. For example, the angular gyrus showed increased FC between the bilateral hippocampus and hippocampal gyrus in the DMN, while decreased FC was observed between the bilateral superior/middle frontal and thalamus in the SN and ECN. These results suggest that the functional allocation of the angular gyrus was increased to compensate the function of the damaged hippocampus, resulting in a relatively reduced load of functions synchronized with the structurally unimpaired areas. This result indicates that structural damage, which is shown mainly in hippocampus and mPFC, can cause functional changes in the triple networks overall by directly or indirectly affecting the function of other areas without structural impairment. The various clinical characteristics associated with the impairment of triple networks in alcoholics may be due to structural damage in just a few areas in these networks, suggesting that the key node of these altered function is the hippocampus.

To examine the pattern of relationship between FC and volume in the control group, we performed the correlation analyses in the ROIs. There is no significant association in these analyses (correlation coefficients: from −0.35 to −0.18, *p*-values: from 0.11 to 0.42). This might reflect lack of significant level of alteration/variability in FC and volume of these areas in the healthy group.

This study has a few limitations. First, the proportion of male participants was significantly higher than that of female participants (~91% for each group). According to previous studies, the prevalence of AUD in men is approximately twice as high as that in women, but the proportion of men who participated in this study was much higher than that in the population. Future studies should include more female participants, since the neurobiological mechanism of AUD in females might be different from those in males (65). Second, the sample size in the study was relatively small (*n* = 20 for each group), which limits the generalizability of the study findings. Nevertheless, the patient sample consisted of well-diagnosed, stable, chronic individuals with AUD, and the sample size provided enough power to detect between-group differences. However, to provide more generalizable insights into PHB, larger sample sizes should be used in future studies. Lastly, because it was a cross-sectional study, we could not determine the causal relationship showing whether the altered FC or structural damage was due to the effect of alcohol, or whether these characteristics led to AUD. However, all the participants were sober for at least 5 months, thus excluding the possibility of active on-going damage by alcohol at the time of study. In addition, previous studies have shown the neuro-degenerative effect of alcohol on the neural areas implicated in the current study (66–69).

Despite these limitations, the current study provided empirical evidence showing that the frontal lobe and hippocampus in the triple network (SN/DMN/ECN) were particularly vulnerable to alcohol. Furthermore, we identified that the increase in FC was related to the brain's compensatory mechanism activated by structural damage. These findings provide future directions for the study of FC and structural alteration in AUDs, as potential biomarkers of disease severity and targets for therapeutic intervention.

## Data Availability Statement

The data that support the findings of this study are available on request from the corresponding author. The data are not publicly available due to restrictions (containing information that could compromise the privacy of research participants).

## Ethics Statement

The studies involving human participants were reviewed and approved by the Institutional Review Board of the Korea Basic Science Institute (IRB approval ID: KBSI-IRB-2017-01). The patients/participants provided their written informed consent to participate in this study.

## Author Contributions

J-WS and CC conceived and planned the experiments and carried out the experiments. J-WS, SH, and CC contributed to the interpretation of the results. J-WS took the lead in writing the manuscript. All authors provided critical feedback, helped shape the research, and analysis and manuscript.

## Funding

This work was supported by the grants from the National Research Foundation of Korea (NRF-2017R1A2B4012546) and the Korea Basic Science Institute (C010300).

## Conflict of Interest

The authors declare that the research was conducted in the absence of any commercial or financial relationships that could be construed as a potential conflict of interest.

## Publisher's Note

All claims expressed in this article are solely those of the authors and do not necessarily represent those of their affiliated organizations, or those of the publisher, the editors and the reviewers. Any product that may be evaluated in this article, or claim that may be made by its manufacturer, is not guaranteed or endorsed by the publisher.
