# Supplementary material for: Functional and Structural Alteration of Default Mode, Executive Control, and Salience Networks in Alcohol Use Disorder
Source: Front Psychiatry. 2021 Oct 20;12:742228. doi: 10.3389/fpsyt.2021.742228 (PMC8564495; doi:10.3389/fpsyt.2021.742228)
Supplement: Supplementary file 1 [file Data_Sheet_1.docx]

Supplementary Material

# Supplemental Material Section 1: 50 pair-wise ROI combinations for functional connectivity analysis

**Anterior salience network:** 1) left middle frontal gyrus – left insula; 2) left middle frontal gyrus – anterior cingulate gyrus, supplementary motor area, medial prefrontal cortex; 3) left middle frontal gyrus – right middle frontal gyrus; 4) left middle frontal gyrus – right insula; 5) left middle frontal gyrus – left lobule VI, Crus I; 6) left middle frontal gyrus – right lobule VI, Crus I; 7) left insula - anterior cingulate gyrus, supplementary motor area, medial prefrontal cortex; 8) left insula - right middle frontal gyrus; 9) left insula - right insula; 10) left insula - left lobule VI, Crus I; 11) left insula - right lobule VI, Crus I; 12) anterior cingulate gyrus, supplementary motor area, medial prefrontal cortex – right middle frontal gyrus; 13) anterior cingulate gyrus, supplementary motor area, medial prefrontal cortex – right insula ;14) anterior cingulate gyrus, supplementary motor area, medial prefrontal cortex - left lobule VI, Crus I; 15) anterior cingulate gyrus, supplementary motor area, medial prefrontal cortex - right lobule VI, Crus I; 16) right middle frontal gyrus - right insula; 17) right middle frontal gyrus - left lobule VI, Crus I; 18) right middle frontal gyrus - right lobule VI, Crus I; 19) right insula – left lobule VI, Crus I; 20) right insula - right lobule VI, Crus I; 21) left lobule VI, Crus I - right lobule VI, Crus I

**Posterior salience network:** 1) left middle frontal gyrus – left supramarginal gyrus, inferior parietal gyrus; 2) left middle frontal gyrus – left precuneus; 3) left middle frontal gyrus – right middle cingulate cortex; 4) left middle frontal gyrus – right superior parietal gyrus, precuneus; 5) left middle frontal gyrus – right supramarginal gyrus, inferior parietal gyrus; 6) left middle frontal gyrus – left thalamus; 7) left middle frontal gyrus – right lobule VI; 8) left middle frontal gyrus – left posterior insula, putamen; 9) left middle frontal gyrus – right thalamus; 10) left middle frontal gyrus – left lobule VI; 11) left middle frontal gyrus – right posterior insula; 12) left supramarginal gyrus, inferior parietal gyrus - left precuneus; 13) left supramarginal gyrus, inferior parietal gyrus - right middle cingulate cortex; 14) left supramarginal gyrus, inferior parietal gyrus - right superior parietal gyrus, precuneus; 15) left supramarginal gyrus, inferior parietal gyrus - right supramarginal gyrus, inferior parietal gyrus; 16) left supramarginal gyrus, inferior parietal gyrus - left thalamus; 17) left supramarginal gyrus, inferior parietal gyrus - right lobule VI; 18) left supramarginal gyrus, inferior parietal gyrus - left posterior insula, putamen; 19) left supramarginal gyrus, inferior parietal gyrus - right thalamus; 20) left supramarginal gyrus, inferior parietal gyrus – left lobule VI; 21) left supramarginal gyrus, inferior parietal gyrus - right posterior insula; 22) left precuneus - right middle cingulate cortex; 23) left precuneus - right superior parietal gyrus, precuneus; 24) left precuneus - right supramarginal gyrus, inferior parietal gyrus; 25) left precuneus - left thalamus; 26) left precuneus - right lobule VI; 27) left precuneus - left posterior insula, putamen; 28) left precuneus - right thalamus; 29) left precuneus – left lobule VI; 30) left precuneus - right posterior insula; 31) right middle cingulate cortex - right superior parietal gyrus, precuneus; 32) right middle cingulate cortex - right supramarginal gyrus, inferior parietal gyrus; 33) right middle cingulate cortex - left thalamus; 34) right middle cingulate cortex - right lobule VI; 35) right middle cingulate cortex - left posterior insula, putamen; 36) right middle cingulate cortex - right thalamus; 37) right middle cingulate cortex - left lobule VI; 38) right middle cingulate cortex - right posterior insula; 39) right superior parietal gyrus, precuneus – right supramarginal gyrus, inferior parietal gyrus; 40) right superior parietal gyrus, precuneus - left thalamus; 41) right superior parietal gyrus, precuneus - right lobule VI; 42) right superior parietal gyrus, precuneus - left posterior insula, putamen; 43) right superior parietal gyrus, precuneus - right thalamus; 44) right superior parietal gyrus, precuneus - left lobule VI; 45) right superior parietal gyrus, precuneus - right posterior insula; 46) right supramarginal gyrus, inferior parietal gyrus - right lobule VI; 47) right supramarginal gyrus, inferior parietal gyrus - left thalamus; 48) right supramarginal gyrus, inferior parietal gyrus - left posterior insula, putamen; 49) right supramarginal gyrus, inferior parietal gyrus - right thalamus; 50) right supramarginal gyrus, inferior parietal gyrus - left lobule VI; 51) right supramarginal gyrus, inferior parietal gyrus – right posterior insula; 52) left thalamus - right lobule VI; 53) left thalamus - left posterior insula, putamen; 54) left thalamus - right thalamus; 55) left thalamus - left lobule VI; 56) left thalamus - right posterior insula; 57) right lobule VI - left posterior insula, putamen; 58) right lobule VI - right thalamus; 59) right lobule VI – right posterior insula; 60) right lobule VI – left lobule VI; 61) left posterior insula, putamen - right thalamus; 62) left posterior insula, putamen - left lobule VI; 63) left posterior insula, putamen - right posterior insula; 64) right thalamus - right posterior insula; 65) right thalamus - left lobule VI; 66) left lobule VI - right posterior insula

**Dorsal Default Mode Network:** 1) Medial prefrontal cortex, anterior cingulate cortex, orbitofrontal cortex – left angular gyrus; 2) Medial prefrontal cortex, anterior cingulate cortex, orbitofrontal cortex - right superior frontal gyrus; 3) Medial prefrontal cortex, anterior cingulate cortex, orbitofrontal cortex - posterior cingulate cortex, precuneus; 4) Medial prefrontal cortex, anterior cingulate cortex, orbitofrontal cortex - midcingulate cortex; 5) Medial prefrontal cortex, anterior cingulate cortex, orbitofrontal cortex - right angular gyrus; 6) Medial prefrontal cortex, anterior cingulate cortex, orbitofrontal cortex - thalamus; 7) Medial prefrontal cortex, anterior cingulate cortex, orbitofrontal cortex - left hippocampus; 8) Medial prefrontal cortex, anterior cingulate cortex, orbitofrontal cortex – right hippocampus; 9) left angular gyrus – right superior frontal gyrus; 10) left angular gyrus – thalamus; 11) left angular gyrus – posterior cingulate cortex, precuneus; 12) left angular gyrus – midcingulate cortex; 13) left angular gyrus – right angular gyrus; 14) left angular gyrus – left hippocampus; 15) left angular gyrus – right hippocampus; 16) right superior frontal gyrus - posterior cingulate cortex, precuneus; 17) right superior frontal gyrus - midcingulate cortex; 18) right superior frontal gyrus - right angular gyrus; 19) right superior frontal gyrus - thalamus; 20) right superior frontal gyrus - left hippocampus; 21) right superior frontal gyrus – right hippocampus; 22) posterior cingulate cortex, precuneus – midcingulate cortex; 23) posterior cingulate cortex, precuneus - right angular gyrus; 24) posterior cingulate cortex, precuneus - thalamus; 25) posterior cingulate cortex, precuneus - left hippocampus; 26) posterior cingulate cortex, precuneus – right hippocampus; 27) midcingulate cortex - right angular gyrus; 28) midcingulate cortex - thalamus; 29) midcingulate cortex - left hippocampus; 30) midcingulate cortex – right hippocampus; 31) right angular gyrus – thalamus; 32) right angular gyrus - left hippocampus; 33) right angular gyrus – right hippocampus; 34) thalamus – left hippocampus; 35) thalamus – right hippocampus; 36) left hippocampus – right hippocampus

**Ventral default mode network:** 1) left restrosplenial cortex, posterior cingulate cortex - left middle frontal gyrus; 2) left restrosplenial cortex, posterior cingulate cortex - left parahippocampal gyrus; 3) left restrosplenial cortex, posterior cingulate cortex - left middle occipital gyrus; 4) left restrosplenial cortex, posterior cingulate cortex - right retrosplenial cortex, Posterior cingulate cortex; 5) left restrosplenial cortex, posterior cingulate cortex – precuneus; 6) left restrosplenial cortex, posterior cingulate cortex - right superior frontal gyrus, middle frontal gyrus; 7) left restrosplenial cortex, posterior cingulate cortex - right parahippocampal gyrus; 8) left restrosplenial cortex, posterior cingulate cortex - right angular gyrus, Middle occipital gyrus; 9) left restrosplenial cortex, posterior cingulate cortex - right lobule IX; 10) left middle frontal gyrus - left parahippocampal gyrus; 11) left middle frontal gyrus - left middle occipital gyrus; 12) left middle frontal gyrus - right retrosplenial cortex, Posterior cingulate cortex; 13) left middle frontal gyrus – precuneus; 14) left middle frontal gyrus - right superior frontal gyrus, middle frontal gyrus; 15) left middle frontal gyrus - right parahippocampal gyrus; 16) left middle frontal gyrus - right angular gyrus, Middle occipital gyrus; 17) left middle frontal gyrus - right lobule IX; 18) left parahippocampal gyrus - left middle occipital gyrus; 19) left parahippocampal gyrus - right retrosplenial cortex, Posterior cingulate cortex; 20) left parahippocampal gyrus – precuneus; 21) left parahippocampal gyrus - right superior frontal gyrus, middle frontal gyrus; 22) left parahippocampal gyrus - right parahippocampal gyrus; 23) left parahippocampal gyrus - right angular gyrus, Middle occipital gyrus; 24) left parahippocampal gyrus - right lobule IX; 25) left middle occipital gyrus - right retrosplenial cortex, Posterior cingulate cortex; 26) left middle occipital gyrus – precuneus; 27) left middle occipital gyrus - right superior frontal gyrus, middle frontal gyrus; 28) left middle occipital gyrus - right parahippocampal gyrus; 29) left middle occipital gyrus - right angular gyrus, Middle occipital gyrus; 30) left middle occipital gyrus - right lobule IX; 31) right retrosplenial cortex, Posterior cingulate cortex – precuneus; 32) right retrosplenial cortex, Posterior cingulate cortex - right superior frontal gyrus, middle frontal gyrus; 33) right retrosplenial cortex, Posterior cingulate cortex - right parahippocampal gyrus; 34) right retrosplenial cortex, Posterior cingulate cortex - right angular gyrus, Middle occipital gyrus; 35) right retrosplenial cortex, Posterior cingulate cortex - right lobule IX; 36) precuneus - right superior frontal gyrus, middle frontal gyrus; 37) precuneus - right parahippocampal gyrus; 38) precuneus - right angular gyrus, Middle occipital gyrus; 39) precuneus - right lobule IX; 40) right superior frontal gyrus, middle frontal gyrus - right parahippocampal gyrus; 41) right superior frontal gyrus, middle frontal gyrus - right angular gyrus, Middle occipital gyrus; 42) right superior frontal gyrus, middle frontal gyrus - right lobule IX; 43) right parahippocampal gyrus - right angular gyrus, Middle occipital gyrus; 44) right parahippocampal gyrus - right lobule IX; 45) right angular gyrus, Middle occipital gyrus – right lobule IX

**Left executive control network:** 1) left middle frontal gyrus, superior frontal gyrus - left inferior frontal gyrus, orbitofrontal gyrus; 2) left middle frontal gyrus, superior frontal gyrus - left superior parietal gyrus, inferior parietal gyrus, precuneus, angular gyrus; 3) left middle frontal gyrus, superior frontal gyrus - left inferior temporal gyrus, middle temporal gyrus; 4) left middle frontal gyrus, superior frontal gyrus - left thalamus; 5) left middle frontal gyrus, superior frontal gyrus - right curs I; 6) left inferior frontal gyrus, orbitofrontal gyrus - left superior parietal gyrus, inferior parietal gyrus, precuneus, angular gyrus; 7) left inferior frontal gyrus, orbitofrontal gyrus - left inferior temporal gyrus, middle temporal gyrus; 8) left inferior frontal gyrus, orbitofrontal gyrus - left thalamus; 9) left inferior frontal gyrus, orbitofrontal gyrus - right curs I; 10) left superior parietal gyrus, inferior parietal gyrus, precuneus, angular gyrus - left inferior temporal gyrus, middle temporal gyrus; 11) left superior parietal gyrus, inferior parietal gyrus, precuneus, angular gyrus - left thalamus; 12) left superior parietal gyrus, inferior parietal gyrus, precuneus, angular gyrus - right curs I; 13) left inferior temporal gyrus, middle temporal gyrus - left thalamus; 14) left inferior temporal gyrus, middle temporal gyrus - right curs I; 15) right curs I – left thalamus

Right executive control network: 1) right middle frontal gyrus, superior frontal gyrus - right middle frontal gyrus; 2) right middle frontal gyrus, superior frontal gyrus - right inferior parietal gyrus, supramarginal gyrus, angular gyrus; 3) right middle frontal gyrus, superior frontal gyrus - right superior frontal gyrus; 4) right middle frontal gyrus, superior frontal gyrus - left crus I/II, lobule VI; 5) right middle frontal gyrus, superior frontal gyrus – right caudate; 6) right middle frontal gyrus - right inferior parietal gyrus, supramarginal gyrus, angular gyrus; 7) right middle frontal gyrus - right superior frontal gyrus; 8) right middle frontal gyrus - left crus I/II, lobule VI; 9) right middle frontal gyrus – right caudate; 10) right inferior parietal gyrus, supramarginal gyrus, angular gyrus - right superior frontal gyrus; 11) right inferior parietal gyrus, supramarginal gyrus, angular gyrus - left crus I/II, lobule VI; 12) right inferior parietal gyrus, supramarginal gyrus, angular gyrus – right caudate; 13) right superior frontal gyrus - left crus I/II, lobule VI; 14) right superior frontal gyrus – right caudate; 15) left crus I/II, lobule VI – right caudate

# Supplemental Figures

# Figure S1. Difference in functional connectivity between the AUD and healthy control groups in the anterior salience network

**
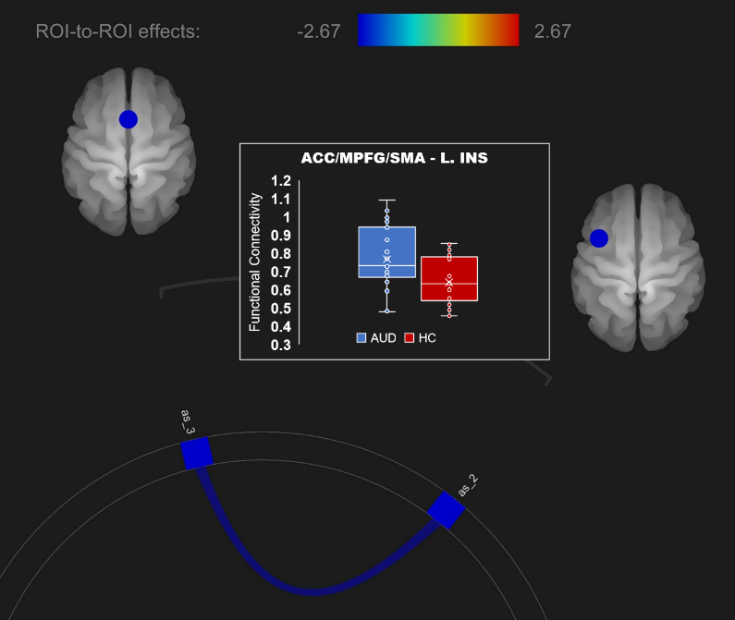
**

Functional connectivity between the left insula and anterior cingulate cortex/supplementary motor area/medial prefrontal cortex (*p* < 0.05, false discovery rate-corrected).

Red and blue squares represent the healthy control and AUD participants, respectively.

**Figure S2. Difference in functional connectivity between the AUD and healthy control groups in the posterior salience network**

**
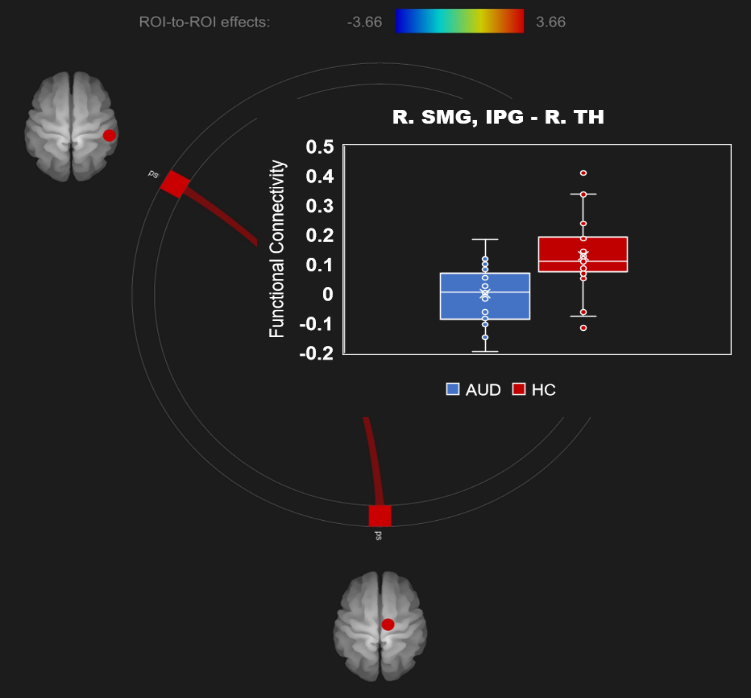
**

Functional connectivity between the right thalamus and left supramarginal gyrus/inferior parietal gyrus (*p* < 0.05, false discovery rate-corrected).

Red and blue squares represent the healthy control and AUD participants, respectively.

**Figure S3. Difference in functional connectivity between the AUD and HC groups in the dorsal default mode network**

**
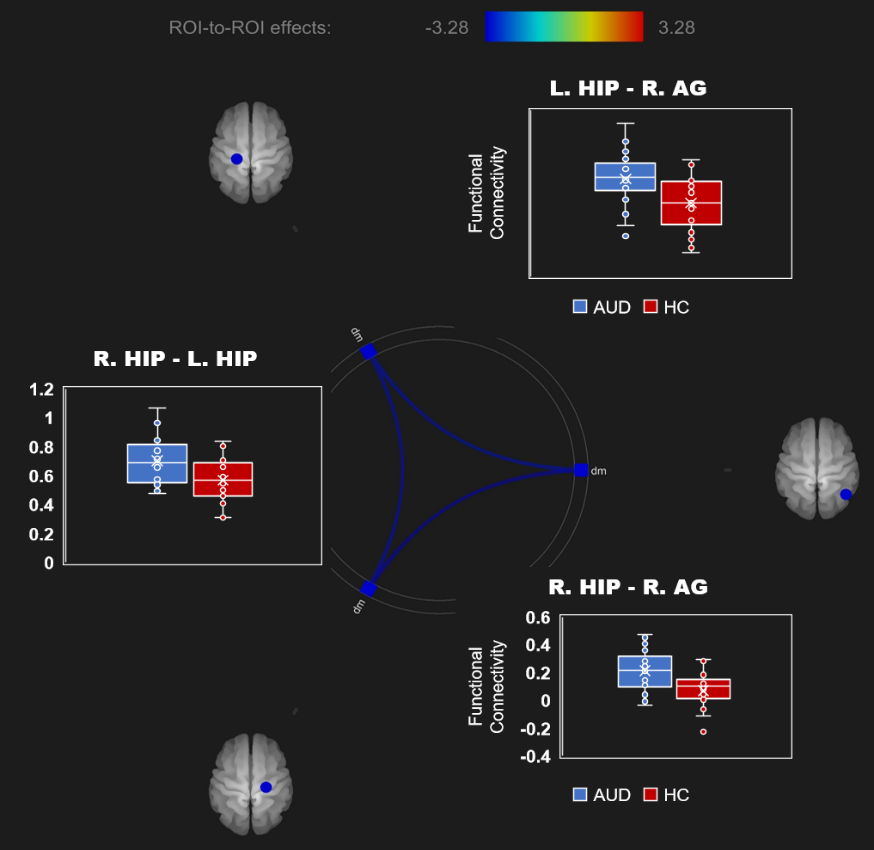
**

Functional connectivity between (1) the right hippocampus and the left hippocampus; (2) the right hippocampus and the right angular gyrus; (3) the left hippocampus and the right angular gyrus (*p* < 0.05, false discovery rate-corrected).

Red and blue squares represent the healthy control and AUD participants, respectively.

**Figure S4. Difference in functional connectivity between the AUD and HC groups in the ventral default mode network**

**
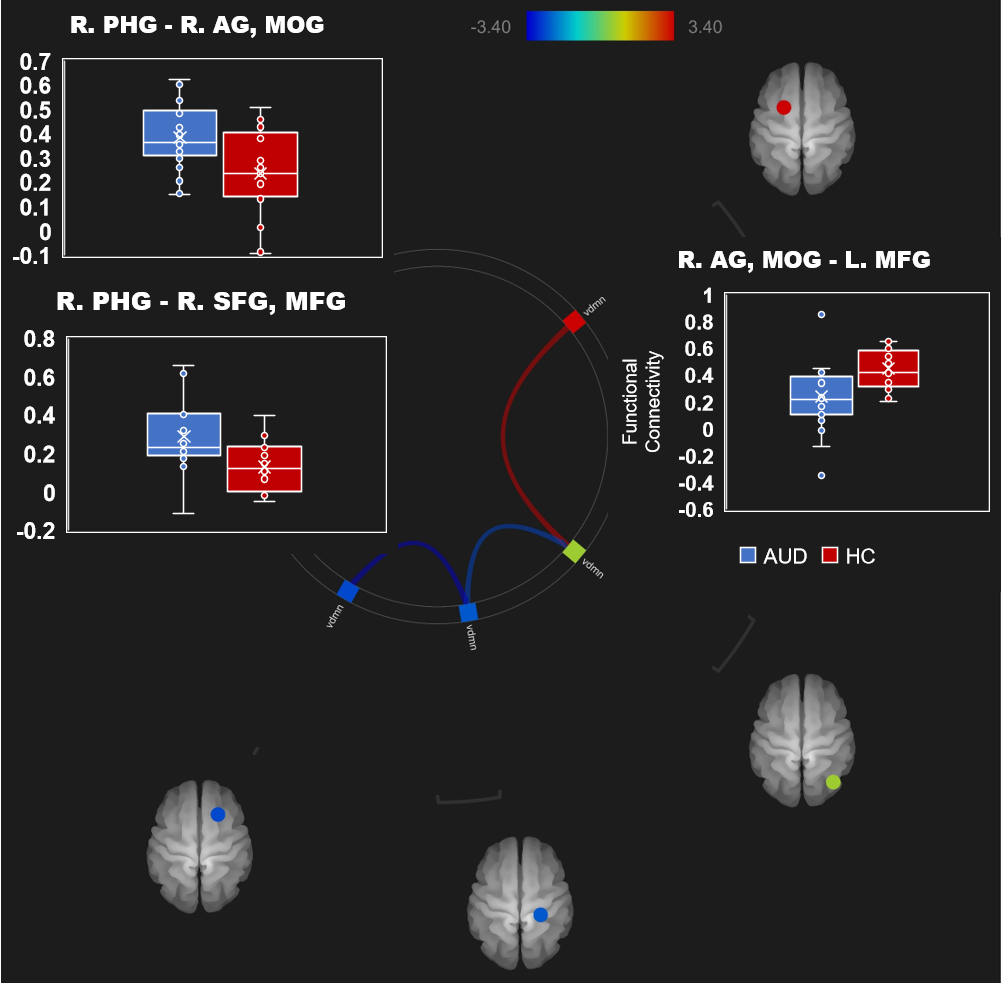
**

Functional connectivity between (1) the right parahippocampal gyrus and the right angular gyrus/middle occipital gyrus; (2) the right parahippocampal gyrus and right superior frontal gyrus and middle frontal gyrus; (3) the right angular gyrus/middle occipital gyrus and left middle frontal gyrus (*p* < 0.05, false discovery rate-corrected).

Red and blue squares represent the healthy control and AUD participants, respectively.

**Figure S5. Difference in functional connectivity between the AUD and HC groups in the**

**right executive control network**

**
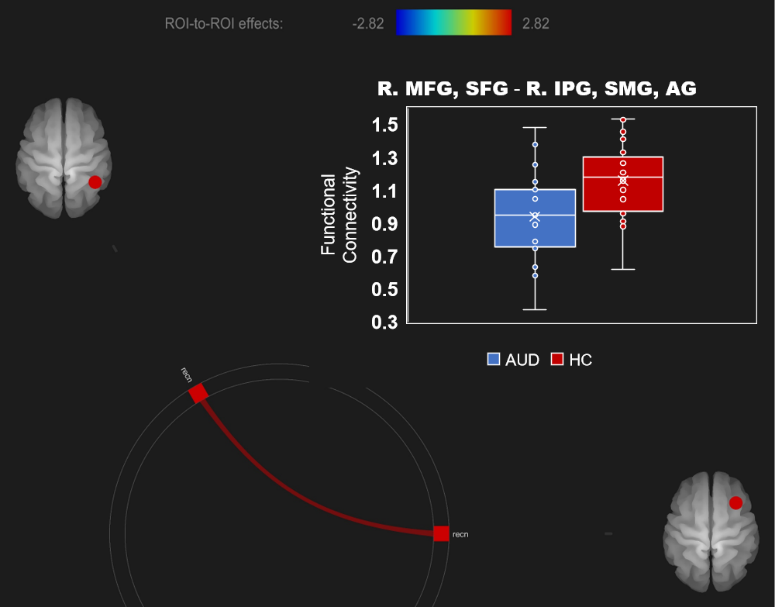
**

Functional connectivity between the right inferior parietal gyrus/supramarginal gyrus/ angular gyrus and right middle frontal gyrus/superior frontal gyrus and (*p* < 0.05, false discovery rate-corrected).

Red and blue squares represent the healthy control and AUD participants, respectively.

# Supplemental Tables

**Table S1. The difference of gray matter volume in anterior salience network between groups**

| **Regions** | **Side** | **BA** | **AUD** | **HC** | **t** |
| --- | --- | --- | --- | --- | --- |
| Middle frontal gyrus | L | 9, 46 | 2.610  (0.358) | 2.750  (0.310) | 1.37 |
| Insula | L | 47, 48 | 1.226  (0.149) | 1.308  (0.129) | 1.90 |
| Anterior cingulate cortex, Medial prefrontal cortex, Supplementary motor area |  | 6, 8,  24, 32 | 10.064  (1.247) | 10.818  (1.003) | ***2.18**** |
| Middle frontal gyrus | R | 9, 46 | 1.969  (0.323) | 2.077  (0.307) | 1.12 |
| Insula | R | 47, 48 | 1.356  (0.205) | 1.459  (1.471) | 1.90 |
| Lobule VI, Crus I | L |  | 0.395  (0.061) | 0.415  (0.062) | 1.12 |
| Lobule VI, Crus I | R |  | 0.558  (0.085) | 0.586  (0.076) | 1.12 |

Abbreviations: AUD, Alcohol Use Disorder group; B, Bilateral; BA, Brodmann Area; HC, Healthy Control group; L, Left; R, Right.

Mean (standard deviation) are presented in AUD and HC columns.

**p<0.05.*

**Table S2. The difference of gray matter volume in posterior salience network between groups**

| **Regions** | **Side** | **BA** | **AUD** | **HC** | **t** |
| --- | --- | --- | --- | --- | --- |
| Middle frontal gyrus | L | 46 | 0.360  (0.067) | 0.346  (0.055) | 0.77 |
| Supramarginal gyrus, Inferior parietal gyrus | L | 40 | 4.103  (0.534) | 4.261  (0.511) | 0.99 |
| Precuneus | L | 5 | 0.353  (0.074) | 0.353  (0.061) | 0.00 |
| Midcingulate cortex | R | 23 | 0.262  (0.047) | 0.291  (0.051) | 1.90 |
| Superior parietal gyrus, Precuneus | R | 5, 7 | 0.457  (0.099) | 0.465  (0.070) | 0.32 |
| Supramarginal gyrus, Inferior parietal gyrus | R | 2, 40 | 3.637  (0.520) | 3.584  (0.399) | 0.38 |
| Thalamus | R |  | 0.684  (0.113) | 0.714  (0.084) | 0.99 |
| Lobule VI | R |  | 0.248  (0.042) | 0.272  (0.051) | 1.63 |
| Posterior insula, Putamen | L | 48 | 0.404  (0.052) | 0.426  (0.036) | 1.59 |
| Thalamus | L |  | 0.340  (0.070) | 0.378  (0.053) | ***2.03**** |
| Lobule VI | L |  | 0.029  (0.009) | 0.034  (0.010) | ***2.10**** |
| Posterior insula | R | 48 | 0.609  (0.090) | 0.676  (0.056) | ***2.95***** |

Abbreviations: AUD, Alcohol Use Disorder group; B, Bilateral; BA, Brodmann Area; HC, Healthy Control group; L, Left; R, Right.

Mean (standard deviation) are presented in AUD and HC columns.

**p<0.05, **p<0.01.*

**Table S3. The difference of gray matter volume in left executive control network between groups**

| **Regions** | **Side** | **BA** | **AUD** | **HC** | **t** |
| --- | --- | --- | --- | --- | --- |
| Middle frontal gyrus, Superior frontal gyrus | L | 8, 9 | 1.566  (0.230) | 1.747  (0.283) | ***2.28**** |
| Inferior frontal gyrus, Orbitofrontal gyrus | L | 10, 45, 47 | 1.285  (0.174) | 1.434  (0.172) | ***2.81***** |
| Superior parietal gyrus, Inferior parietal gyrus, Precuneus, Angular gyrus | L | 7, 39, 40 | 0.025  (0.005) | 0.027  (0.003) | 1.64 |
| Inferior temporal gyrus, Middle temporal gyrus | L | 20, 37 | 5.130  (0.638) | 5.391  (0.529) | 1.46 |
| Crus I | R |  | 1.340  (0.177) | 1.419  (0.165) | 1.51 |
| Thalamus | L |  | 6.197  (0.749) | 6.250  (0.782) | 0.23 |

Abbreviations: AUD, Alcohol Use Disorder group; B, Bilateral; BA, Brodmann Area; HC, Healthy Control group; L, Left; R, Right.

Mean (standard deviation) are presented in AUD and HC columns.

**p<0.05, **p<0.01.*

**Table S4. The difference of gray matter volume in right executive control network between groups**

| **Regions** | **Side** | **BA** | **AUD** | **HC** | **t** |
| --- | --- | --- | --- | --- | --- |
| Middle frontal gyrus, Superior frontal gyrus | R | 8, 9, 46 | 6.729  (0.827) | 7.127  (0.505) | 1.90 |
| Middle frontal gyrus | R | 10, 46 | 1.546  (0.236) | 1.591  (0.206) | 0.68 |
| Inferior parietal gyrus, Supramarginal gyrus, angular gyrus | R | 7, 39, 40 | 5.323  (0.756) | 5.558  (0.578) | 1.14 |
| Superior frontal gyrus | R | 8 | 0.273  (0.041) | 0.289  (0.034) | 1.37 |
| Crus I, II, Lobule VI | L |  | 8.849  (0.935) | 9.605  (1.106) | ***2.40**** |
| Caudate | R |  | 0.685  (0.097) | 0.698  (0.076) | 0.46 |

Abbreviations: AUD, Alcohol Use Disorder group; B, Bilateral; BA, Brodmann Area; HC, Healthy Control group; L, Left; R, Right.

Mean (standard deviation) are presented in AUD and HC columns.

**p<0.05.*

**Table S5. The difference of gray matter volume in dorsal default mode network between groups**

| Regions | Side | BA | AUD | HC | t |
| --- | --- | --- | --- | --- | --- |
| Medial prefrontal cortex, Anterior cingulate cortex, Orbitofrontal cortex |  | 9, 10, 11, 24, 32 | 18.880  (2.036) | 19.983  (1.565) | ***2.12**** |
| Angular gyrus | L | 39 | 0.381  (0.062) | 0.346  (0.068) | 1.75 |
| Superior frontal gyrus | R | 9 | 0.622  (0.112) | 0.641  (0.115) | 0.55 |
| Posterior cingulate cortex, Precuneus |  | 23, 30 | 6.525  (1.004) | 6.528  (0.077) | 0.01 |
| Midcingulate cortex |  | 23 | 0.501  (0.084) | 0.539  (0.070) | 1.62 |
| Angular gyrus | R | 39 | 0.191  (0.039) | 0.203  (0.026) | 1.22 |
| Thalamus |  |  | 0.827  (0.145) | 0.911  (0.149) | 1.88 |
| Hippocampus | L | 20, 30, 36 | 1.421  (0.091) | 1.516  (0.119) | ***2.90***** |
| Hippocampus | R | 20, 30, 36 | 0.479  (0.037) | 0.525  (0.045) | ***2.83***** |

Abbreviations: AUD, Alcohol Use Disorder group; B, Bilateral; BA, Brodmann Area; HC, Healthy Control group; L, Left; R, Right.

Mean (standard deviation) are presented in AUD and HC columns.

**p<0.05, **p<0.01.*

**Table S6. The difference of gray matter volume in ventral default mode network between groups**

| Regions | Side | BA | AUD | HC | t |
| --- | --- | --- | --- | --- | --- |
| Restrosplenial cortex, Posterior cingulate cortex | L | 23, 29, 30 | 1.856  (0.288) | 2.009  (0.245) | 1.81 |
| Middle frontal gyrus | L | 6, 8 | 1.514  (0.211) | 1.560  (0.260) | 1.18 |
| Parahippocampal gyrus | L | 20, 37 | 0.695  (0.076) | 0.768  (0.086) | ***2.94***** |
| Middle occipital gyrus | L | 19, 39 | 1.643  (0.260) | 1.564  (0.221) | 1.08 |
| Retrosplenial cortex, Posterior cingulate cortex | R | 23, 30 | 2.224  (0.336) | 2.353  (0.265) | 1.41 |
| Precuneus |  | 5, 7 | 7.069  (1.084) | 7.062  (0.885) | 0.02 |
| Superior frontal gyrus, Middle frontal gyrus | R | 8, 9 | 1.573  (0.172) | 1.657  (0.196) | 1.49 |
| Parahippocampal gyrus | R | 30, 37 | 0.399  (0.047) | 0.437  (0.047) | ***2.63**** |
| Angular gyrus, Middle occipital gyrus | R | 19, 39 | 2.179  (0.417) | 2.248  (0.299) | 0.62 |
| Lobule IX | R |  | 0.296  (0.048) | 0.328  (0.048) | ***2.10**** |

Abbreviations: AUD, Alcohol Use Disorder group; B, Bilateral; BA, Brodmann Area; HC, Healthy Control group; L, Left; R, Right.

Mean (standard deviation) are presented in AUD and HC columns.

**p<0.05, **p<0.01.*

**Table S7. Correlation coefficients between regions among anterior salience network in each group**

| **Areas** | **Groups** | **L. MFG** | **L. INS** | **ACC, SMA, MPFC** | **R. MFG** | **R. INS** | **L. LVI, CI** |
| --- | --- | --- | --- | --- | --- | --- | --- |
| **L. INS** | AUD | 0.60^***^ |  |  |  |  |  |
|  | HC | 0.59^***^ |  |  |  |  |  |
| **ACC, SMA, MPFC** | AUD | 0.58^***^ | 0.77^***^ |  |  |  |  |
|  | HC | 0.58^***^ | 0.64^***^ |  |  |  |  |
| **R. MFG** | AUD | 0.66^***^ | 0.39^***^ | 0.46^***^ |  |  |  |
|  | HC | 0.63^***^ | 0.39^***^ | 0.51^***^ |  |  |  |
| **R. INS** | AUD | 0.47^***^ | 0.86^***^ | 0.68^***^ | 0.54^***^ |  |  |
|  | HC | 0.48^***^ | 0.81^***^ | 0.66^***^ | 0.51^***^ |  |  |
| **L. LVI, CI** | AUD | 0.35^***^ | 0.27^**^ | 0.34^***^ | 0.35^***^ | 0.30^**^ |  |
|  | HC | 0.34^***^ | 0.18^*^ | 0.27^**^ | 0.37^***^ | 0.23^**^ |  |
| **R. LVI, CI** | AUD | 0.29^***^ | 0.19^**^ | 0.28^**^ | 0.15^*^ | 0.10 | 0.50^***^ |
|  | HC | 0.26^***^ | 0.15^*^ | 0.26^**^ | 0.22^**^ | 0.10 | 0.50^***^ |

Abbreviations: ACC, Anterior Cingulate Cortex; AUD, Alcohol Use Disorder group; HC, Healthy Control group; INS, Insula; CI, Crus I; L, Left; LVI, Lobule VI; MFG, Middle Frontal Gyrus; MPFC, Medial Prefrontal Cortex; R, Right; SMA, Supplementary Motor Area.

Correlation coefficients (Beta values) are presented.

**p<0.05, **p<0.01, and ***p<0.001 (p-FDR, seed-level correction).*

**Table S8. Correlation coefficients between regions among posterior salience network in each group**

| **Areas** | **Groups** | **L. MFG** | **L. SMG, IPG** | **L. PC** | **R. MCC** | **R. SPG, PC** | **R. SMG, IPG** | **L. Th** | **R. LVI** | **L. PI, Pu** | **R. Th** | **L. LVI** |
| --- | --- | --- | --- | --- | --- | --- | --- | --- | --- | --- | --- | --- |
| **L. SMG, IPG** | **AUD** | 0.47^***^ |  |  |  |  |  |  |  |  |  |  |
|  | **HC** | 0.47^***^ |  |  |  |  |  |  |  |  |  |  |
| **L. PC** | **AUD** | 0.04 | 0.21^***^ |  |  |  |  |  |  |  |  |  |
|  | **HC** | -0.03 | 0.07 |  |  |  |  |  |  |  |  |  |
| **R. MCC** | **AUD** | -0.01 | 0.15^***^ | 0.26^***^ |  |  |  |  |  |  |  |  |
|  | **HC** | 0.02 | 0.21^***^ | 0.28^***^ |  |  |  |  |  |  |  |  |
| **R. SPG, PC** | **AUD** | -0.03 | 0.10^**^ | 0.31^***^ | 0.20^***^ |  |  |  |  |  |  |  |
|  | **HC** | -0.04 | 0.01 | 0.44^***^ | 0.27^***^ |  |  |  |  |  |  |  |
| **R. SMG, IPG** | **AUD** | 0.32^***^ | 0.96^***^ | 0.19^**^ | 0.24^***^ | 0.19^**^ |  |  |  |  |  |  |
|  | **HC** | 0.22^***^ | 0.89^***^ | 0.15^**^ | 0.40^***^ | 0.17^**^ |  |  |  |  |  |  |
| **L. Th** | **AUD** | 0.01 | 0.05 | 0.06 | 0.10^*^ | 0.02 | 0.00 |  |  |  |  |  |
|  | **HC** | 0.07 | 0.06 | 0.01 | 0.07 | 0.03 | 0.05 |  |  |  |  |  |
| **R. LVI** | **AUD** | 0.03 | 0.12^**^ | 0.14^**^ | 0.09 | 0.14^**^ | 0.21^***^ | -0.02 |  |  |  |  |
|  | **HC** | -0.06 | 0.04 | 0.04 | 0.10^*^ | 0.13^**^ | 0.14^**^ | -0.06 |  |  |  |  |
| **L. PI, Pu** | **AUD** | 0.06 | 0.34^***^ | 0.16^***^ | 0.26^***^ | 0.15^**^ | 0.41^***^ | 0.07 | 0.06 |  |  |  |
|  | **HC** | 0.04 | 0.38^***^ | 0.20^***^ | 0.35^***^ | 0.17^***^ | 0.50^***^ | 0.12^*^ | 0.06 |  |  |  |
| **R. Th** | **AUD** | -0.01 | 0.00 | 0.02 | 0.08 | -0.02 | 0.00 | 0.59^***^ | -0.03 | 0.06 |  |  |
|  | **HC** | 0.05 | 0.09 | -0.07 | 0.05 | -0.01 | 0.13^*^ | 0.47^***^ | 0.05 | 0.08 |  |  |
| **L. LVI** | **AUD** | 0.01 | -0.02 | 0.12^**^ | -0.01 | 0.04 | -0.01 | -0.01 | 0.15^**^ | -0.06 | -0.01 |  |
|  | **HC** | 0.03 | 0.00 | 0.07 | 0.02 | 0.09 | -0.01 | 0.04 | 0.18^**^ | -0.05 | 0.01 |  |
| **R.**  **PI** | **AUD** | 0.00 | 0.22^***^ | 0.07 | 0.21^***^ | 0.12^*^ | 0.35^***^ | 0.04 | 0.00 | 0.54^***^ | 0.05 | -0.10 |
|  | **HC** | -0.02 | 0.24^***^ | 0.18 | 0.32^***^ | 0.17 | 0.45^***^ | 0.06 | 0.03 | 0.65^***^ | 0.06 | -0.04 |

Abbreviations: AUD, Alcohol Use Disorder group; HC, Healthy Control group; IPG, Inferior Parietal Gyrus; L, Left; LVI, Lobule VI; MCC, Midcingulate cortex; MFG, Middle Frontal Gyrus; PC, Precuneus; R, Right; PI Posterior Insula; Pu, Putamen; SMG, Supramarginal gyrus; SPG, Superior Parietal Gyrus; TH, Thalamus.

Correlation coefficients (Beta values) are presented.

**p<0.05, **p<0.01, and ***p<0.001 (p-FDR, seed-level correction).*

**Table S9. Correlation coefficients between regions among left executive control network in each group**

| **Areas** | **Groups** | **L. MFG, SFG** | **L. IFG, OFG** | **L. SPG, IPG, PC, AG** | **L. ITG, MTG** | **R. CI** |
| --- | --- | --- | --- | --- | --- | --- |
| **L. IFG, OFG** | AUD | 0.49^***^ |  |  |  |  |
|  | HC | 0.50^***^ |  |  |  |  |
| **L. SPG, IPG, PC, AG** | AUD | 0.95^***^ | 0.61^***^ |  |  |  |
|  | HC | 1.00^***^ | 0.56^***^ |  |  |  |
| **L. ITG, MTG** | AUD | 0.50^***^ | 0.50^***^ | 0.65^***^ |  |  |
|  | HC | 0.55^***^ | 0.46^***^ | 0.55^***^ |  |  |
| **R. CI** | AUD | 0.58^***^ | 0.35^***^ | 0.54^***^ | 0.37^***^ |  |
|  | HC | 0.61^***^ | 0.33^***^ | 0.58^***^ | 0.34^***^ |  |
| **L. Th** | AUD | 0.02 | -0.01 | 0.02 | 0.05 | -0.01 |
|  | HC | 0.03 | 0.05 | 0.04 | 0.03 | 0.04 |

Abbreviations: AG, Angular Gyrus; AUD, Alcohol Use Disorder group; HC, Healthy Control group; IFG, Inferior frontal gyrus; IPG, Inferior Parietal Gyrus; ITG, Inferior Temporal Gyrus; CI, Crus I; L, Left; MFG, Middle Frontal Gyrus; MTG, Middle Temporal gyrus; OFG, Orbitofrontal gyrus; PC, Precuneus; R, Right; SFG, Superior Frontal gyrus; SPG, Superior Parietal Gyrus; Th, Thalamus.

Correlation coefficients (Beta values) are presented.

**p<0.05, **p<0.01, and ***p<0.001 (p-FDR, seed-level correction).*

**Table S10. Correlation coefficients between regions among right executive control network in each group**

| **Areas** | **Groups** | **R. MFG, SFG** | **R. MFG** | **R. IPG, SMG, AG** | **R. SFG** | **L. LVI, CI, CII** |
| --- | --- | --- | --- | --- | --- | --- |
| **R. MFG** | AUD | 0.49^***^ |  |  |  |  |
|  | HC | 0.66^***^ |  |  |  |  |
| **R. IPG, SMG, AG** | AUD | 0.94^***^ | 0.56^***^ |  |  |  |
|  | HC | 1.16^***^ | 0.63^***^ |  |  |  |
| **R. SFG** | AUD | 0.43^***^ | 0.26^***^ | 0.39^***^ |  |  |
|  | HC | 0.54^***^ | 0.46^***^ | 0.42^***^ |  |  |
| **L. LVI, CI, CII** | AUD | 0.56^***^ | 0.32^***^ | 0.45^***^ | 0.17^***^ |  |
|  | HC | 0.63^***^ | 0.42^***^ | 0.48^***^ | 0.26^***^ |  |
| **R. Cau** | AUD | 0.19^***^ | 0.08 | 0.12^**^ | 0.15^***^ | 0.11^*^ |
|  | HC | 0.25^***^ | 0.17^***^ | 0.19^***^ | 0.22^***^ | 0.23^***^ |

Abbreviations: AG, Angular Gyrus; AUD, Alcohol Use Disorder group; Cau, Caudate; HC, Healthy Control group; IPG, Inferior Parietal Gyrus; CI, Crus I; CII, Crus II; L, Left; LVI, Lobule VI; MFG, Middle Frontal Gyrus; R, Right; SFG, Superior Frontal gyrus; SMG, Supramarginal Gyrus.

Correlation coefficients (Beta values) are presented.

**p<0.05, **p<0.01, and ***p<0.001 (p-FDR, seed-level correction).*

**Table S11. Correlation coefficients between regions among dorsal default mode network in each group**

| **Areas** | **Groups** | **MPFC, ACC, OFC** | **L. AG** | **R. SFG** | **PCC, PC** | **MCC** | **R. AG** | **Th** | **L. Hip** |
| --- | --- | --- | --- | --- | --- | --- | --- | --- | --- |
| **L. AG** | AUD | 0.54^***^ |  |  |  |  |  |  |  |
|  | HC | 0.43^***^ |  |  |  |  |  |  |  |
| **R. SFG** | AUD | 0.53^***^ | 0.44^***^ |  |  |  |  |  |  |
|  | HC | 0.52^***^ | 0.44^***^ |  |  |  |  |  |  |
| **PCC, PC** | AUD | 0.67^***^ | 0.58^***^ | 0.53^***^ |  |  |  |  |  |
|  | HC | 0.57^***^ | 0.59^***^ | 0.55^***^ |  |  |  |  |  |
| **MCC** | AUD | 0.47^***^ | 0.28^***^ | 0.35^***^ | 0.41^***^ |  |  |  |  |
|  | HC | 0.44^***^ | 0.22^**^ | 0.22^**^ | 0.30^***^ |  |  |  |  |
| **R. AG** | AUD | 0.37^***^ | 0.56^***^ | 0.50^***^ | 0.65^***^ | 0.30^***^ |  |  |  |
|  | HC | 0.27^**^ | 0.53^***^ | 0.50^***^ | 0.48^***^ | 0.22^**^ |  |  |  |
| **Th** | AUD | 0.21^**^ | 0.08 | 0.11 | 0.17^*^ | 0.13^*^ | 0.09 |  |  |
|  | HC | 0.15^*^ | 0.09 | 0.08 | 0.13^*^ | 0.18^*^ | 0.09 |  |  |
| **L. Hip** | AUD | 0.30^***^ | 0.20^**^ | 0.18^*^ | 0.32^***^ | 0.17^*^ | 0.21^**^ | 0.12^*^ |  |
|  | HC | 0.23^**^ | 0.15^*^ | 0.16^*^ | 0.30^***^ | 0.18^*^ | 0.07 | 0.17^*^ |  |
| **R. Hip** | AUD | 0.28^**^ | 0.16^*^ | 0.17^*^ | 0.31^***^ | 0.18^*^ | 0.23^**^ | 0.09 | 0.70^***^ |
|  | HC | 0.18^*^ | 0.08 | 0.12^*^ | 0.19^**^ | 0.11 | 0.10 | 0.12^*^ | 0.57^***^ |

Abbreviations: ACC, Anterior Cingulate Cortex; AG, Angular Gyrus; AUD, Alcohol Use Disorder group; HC, Healthy Control group; Hip, Hippocampus; L, Left; MCC, Midcingulate Cortex; MPFC, Medial Prefrontal Cortex; OFC, Orbitofrontal cortex; PC, Precuneus; PCC, Posterior Cingulate Cortex; R, Right; SFG, Superior Frontal gyrus; Th, Thalamus.

Correlation coefficients (Beta values) are presented.

**p<0.05, **p<0.01, and ***p<0.001 (p-FDR, seed-level correction).*

**Table S12. Correlation coefficients between regions among ventral default mode network in each group**

| **Areas** | **Groups** | **L. RC, PCC** | **L. MFG** | **L. PHG** | **L. MOG** | **R. RC, PCC** | **PC** | **R. SFG, MFG** | **R. PHG** | **R. AG, MOG** |
| --- | --- | --- | --- | --- | --- | --- | --- | --- | --- | --- |
| **L. MFG** | AUD | ^0.14^ |  |  |  |  |  |  |  |  |
|  | HC | ^0.12*^ |  |  |  |  |  |  |  |  |
| **L. PHG** | AUD | ^0.60***^ | ^0.14*^ |  |  |  |  |  |  |  |
|  | HC | ^0.53***^ | ^0.15*^ |  |  |  |  |  |  |  |
| **L. MOG** | AUD | ^0.49***^ | ^0.33***^ | ^0.45***^ |  |  |  |  |  |  |
|  | HC | ^0.39***^ | ^0.45***^ | ^0.39***^ |  |  |  |  |  |  |
| **R. RC, PCC** | AUD | ^1.32***^ | ^0.11^ | ^0.61***^ | ^0.50***^ |  |  |  |  |  |
|  | HC | ^1.24***^ | ^0.14**^ | ^0.57***^ | ^0.41***^ |  |  |  |  |  |
| **PC** | AUD | ^0.41***^ | ^0.33***^ | ^0.31***^ | ^0.45***^ | ^0.51***^ |  |  |  |  |
|  | HC | ^0.46***^ | ^0.41***^ | ^0.34***^ | ^0.46***^ | ^0.56***^ |  |  |  |  |
| **R. SFG, MFG** | AUD | ^0.42***^ | ^0.46***^ | ^0.37***^ | ^0.49***^ | ^0.45***^ | ^0.50***^ |  |  |  |
|  | HC | ^0.32***^ | ^0.52***^ | ^0.27**^ | ^0.44***^ | ^0.37***^ | ^0.51***^ |  |  |  |
| **R. PHG** | AUD | ^0.47***^ | ^0.08*^ | ^0.71***^ | ^0.39***^ | ^0.50***^ | ^0.29***^ | ^0.29***^ |  |  |
|  | HC | ^0.38***^ | ^0.05^ | ^0.60***^ | ^0.26**^ | ^0.43***^ | ^0.21**^ | ^0.13*^ |  |  |
| **R. AG, MOG** | AUD | ^0.46***^ | ^0.24**^ | ^0.43***^ | ^0.85***^ | ^0.55***^ | ^0.52***^ | ^0.67***^ | ^0.38***^ |  |
|  | HC | ^0.33***^ | ^0.45***^ | ^0.34***^ | ^0.79***^ | ^0.43***^ | ^0.48***^ | ^0.64***^ | ^0.23**^ |  |
| **R. LIX** | AUD | ^0.08^ | ^0.02^ | ^0.07^ | ^0.06^ | ^0.08^ | ^0.15*^ | ^0.03^ | ^0.07^ | ^0.06^ |
|  | HC | ^0.01^ | ^0.04^ | ^0.01^ | ^0.06^ | ^0.03^ | ^0.02^ | ^0.02^ | ^0.04^ | ^0.02^ |

Abbreviations: AG, Angular Gyrus; AUD, Alcohol Use Disorder group; HC, Healthy Control group; L, Left; LIX, Lobule IX; MFG, Middle Frontal Gyrus; MOG, Middle Occipital Gyrus; PC, Precuneus; PCC, Posterior Cingulate Cortex; PHG, Parahippocampal Gyrus; R, Right; RC, Restrosplenial Cortex; SPG, Superior Frontal Gyrus.

Correlation coefficients (Beta values) are presented.

**p<0.05, **p<0.01, and ***p<0.001 (p-FDR, seed-level correction).*

**Table S13. Group differences of functional connectivity in anterior salience network**

| **Areas** | **L. MFG** | **L. INS** | **ACC, SMA, MPFC** | **R. MFG** | **R. INS** | **L. LVI, CI** |
| --- | --- | --- | --- | --- | --- | --- |
| **L. INS** | 0.10 |  |  |  |  |  |
| **ACC, SMA, MPFC** | 0.05 | **2.67*** |  |  |  |  |
| **R. MFG** | 0.40 | 0.06 | 0.67 |  |  |  |
| **R. INS** | 0.09 | 0.64 | 0.45 | 0.34 |  |  |
| **L. LVI, CI** | 0.19 | 1.58 | 1.12 | 0.31 | 1.18 |  |
| **R. LVI, CI** | 0.57 | 0.91 | 0.33 | 1.45 | 0.06 | 0.04 |

Abbreviations: ACC, Anterior Cingulate Cortex; AUD, Alcohol Use Disorder group; HC, Healthy Control group; INS, Insula; CI, Crus I; L, Left; LVI, Lobule VI; MFG, Middle Frontal Gyrus; MPFC, Medial Prefrontal Cortex; R, Right; SMA, Supplementary Motor Area.

t values are presented.

**p<0.05.*

**Table S14. Group differences of functional connectivity in posterior salience network**

| **Areas** | **L. MFG** | **L. SMG, IPG** | **L. PC** | **R. MCC** | **R. SPG, PC** | **R. SMG, IPG** | **L. Th** | **R. LVI** | **L. PI, Pu** | **R. Th** | **L. LVI** |
| --- | --- | --- | --- | --- | --- | --- | --- | --- | --- | --- | --- |
| **L. SMG, IPG** | 0.02 |  |  |  |  |  |  |  |  |  |  |
| **L. PC** | 1.03 | 2.08 |  |  |  |  |  |  |  |  |  |
| **R. MCC** | -0.59 | -0.89 | -0.30 |  |  |  |  |  |  |  |  |
| **R. SPG, PC** | 0.09 | 1.66 | -1.76 | -1.31 |  |  |  |  |  |  |  |
| **R. SMG, IPG** | 1.06 | 0.98 | 0.56 | -1.94 | 0.30 |  |  |  |  |  |  |
| **L. Th** | -1.39 | -0.28 | 1.16 | 0.50 | -0.21 | -1.11 |  |  |  |  |  |
| **R. LVI** | 1.71 | 1.66 | 2.15 | -0.06 | 0.25 | 1.50 | 1.09 |  |  |  |  |
| **L. PI, Pu** | 0.34 | -0.52 | -0.86 | 1.65 | -0.35 | -1.21 | -1.13 | -0.10 |  |  |  |
| **R. Th** | -1.44 | -2.59 | 1.74 | 0.61 | -0.19 | ***-3.66^**^*** | 2.08 | -2.30 | -0.51 |  |  |
| **L. LVI** | -0.46 | -0.42 | 1.52 | -0.92 | -1.23 | 0.02 | -1.23 | -0.55 | -0.50 | -0.57 |  |
| **R.**  **PI** | 0.16 | -0.32 | -2.18 | -2.00 | -0.88 | -1.20 | -0.52 | -0.63 | -2.01 | -0.06 | -1.72 |

Abbreviations: AUD, Alcohol Use Disorder group; HC, Healthy Control group; IPG, Inferior Parietal Gyrus; L, Left; LVI, Lobule VI; MCC, Midcingulate cortex; MFG, Middle Frontal Gyrus; PC, Precuneus; R, Right; PI Posterior Insula; Pu, Putamen; SMG, Supramarginal gyrus; SPG, Superior Parietal Gyrus; Th, Thalamus.

t values are presented.

**p<0.05.*

**Table S15. Group differences of functional connectivity in left executive control network**

| **Areas** | **L. MFG, SFG** | **L. IFG, OFG** | **L. SPG, IPG, PC, AG** | **L. ITG, MTG** | **R. CI** |
| --- | --- | --- | --- | --- | --- |
| **L. IFG, OFG** | -0.21 |  |  |  |  |
| **L. SPG, IPG, PC, AG** | -0.74 | 0.61 |  |  |  |
| **L. ITG, MTG** | -0.68 | 0.70 | 1.38 |  |  |
| **R. CI** | -0.49 | 0.20 | 0.56 | 0.53 |  |
| **L. Th** | -0.17 | 1.55 | 0.34 | 0.57 | 1.05 |

Abbreviations: AG, Angular gyrus; AUD, Alcohol Use Disorder group; HC, Healthy Control group; CI, Crus I; IFG, Inferior frontal gyrus; ITG, Inferior temporal gyrus; L, Left; MFG, Middle Frontal Gyrus; MTG, middle temporal gyrus; PC, precuneus; R, Right; SFG, Superior frontal cortex; SPG, superior parietal gyrus.

t values are presented.

**Table S16. Group differences of functional connectivity in right executive control network**

|  | **R. MFG, SFG** | **R. MFG** | **R. IPG, SMG, AG** | **R. SFG** | **L. LVI, CI, CII** |
| --- | --- | --- | --- | --- | --- |
| **R. MFG** | -1.81 |  |  |  |  |
| **R. IPG, SMG, AG** | **-2.82^*^** | -0.85 |  |  |  |
| **R. SFG** | -1.58 | -2.37 | -0.41 |  |  |
| **L. LVI, CI, CII** | -0.94 | -1.52 | -0.50 | -1.41 |  |
| **R. Cau** | -1.33 | -1.82 | -1.37 | -1.64 | -2.13 |

Abbreviations: AG, Angular gyrus; AUD, Alcohol Use Disorder group; HC, Healthy Control group; CI, Crus I; CII, Crus II; L, Left; LVI, Lobule VI; MFG, Middle Frontal Gyrus; R, Right; SFG, Superior frontal gyrus; SMG, Supramarginal gyrus.

t values are presented.

**p<0.05.*

**Table S17. Group differences of functional connectivity in dorsal default mode network**

|  | **MPFC, ACC, OFC** | **L. AG** | **R. SFG** | **PCC, PC** | **MCC** | **R. AG** | **Th** | **L. Hip** |
| --- | --- | --- | --- | --- | --- | --- | --- | --- |
| **L. AG** | 1.74 |  |  |  |  |  |  |  |
| **R. SFG** | 0.05 | 0.09 |  |  |  |  |  |  |
| **PCC, PC** | 1.48 | -0.17 | -0.24 |  |  |  |  |  |
| **MCC** | 0.50 | 0.92 | 2.43 | 1.50 |  |  |  |  |
| **R. AG** | 1.51 | 0.41 | 0.00 | 2.11 | 1.40 |  |  |  |
| **Th** | 1.36 | -0.15 | 0.74 | 1.11 | -1.10 | 0.14 |  |  |
| **L. Hip** | 1.34 | 0.71 | 0.48 | 0.39 | -0.25 | **2.92^*^** | -0.94 |  |
| **R. Hip** | 1.73 | 1.34 | 0.98 | 2.14 | 1.54 | **3.28^*^** | -0.56 | **2.91^*^** |

Abbreviations: ACC, Anterior Cingulate Cortex; AG, Angular Gyrus; AUD, Alcohol Use Disorder group; HC, Healthy Control group; Hip, Hippocampus; L, Left; MCC, Midcingulate Cortex; MPFC, Medial Prefrontal Cortex; OFC, Orbitofrontal cortex; PC, Precuneus; PCC, Posterior Cingulate Cortex; R, Right; SFG, Superior Frontal gyrus; Th, Thalamus.

t values are presented.

**p<0.05.*

**Table S18. Group differences of functional connectivity in ventral default mode network**

| **Areas** | **L. RC, PCC** | **L. MFG** | **L. PHG** | **L. MOG** | **R. RC, PCC** | **PC** | **R. SFG, MFG** | **R. PHG** | **R. AG, MOG** |
| --- | --- | --- | --- | --- | --- | --- | --- | --- | --- |
| **L. MFG** | 0.16 |  |  |  |  |  |  |  |  |
| **L. PHG** | 0.90 | -0.22 |  |  |  |  |  |  |  |
| **L. MOG** | 1.01 | -1.50 | 0.94 |  |  |  |  |  |  |
| **R. RC, PCC** | 0.97 | -0.45 | 0.64 | 1.16 |  |  |  |  |  |
| **PC** | -0.55 | -1.28 | -0.48 | -0.19 | -0.68 |  |  |  |  |
| **R. SFG, MFG** | 1.19 | -0.84 | 1.53 | 0.77 | 1.09 | -0.19 |  |  |  |
| **R. PHG** | 1.21 | 0.76 | 1.67 | 2.33 | 0.99 | 1.29 | **3.34^*^** |  |  |
| **R. AG, MOG** | 1.51 | **-3.40^*^** | 1.47 | 0.75 | 1.56 | 0.53 | 0.38 | **3.09*** |  |
| **R. LIX** | 1.80 | -0.43 | 1.64 | 0.01 | 1.45 | **3.27^*^** | 0.46 | 0.93 | 0.92 |

Abbreviations: AG, Angular Gyrus; AUD, Alcohol Use Disorder group; HC, Healthy Control group; L, Left; LIX, Lobule IX; MFG, Middle Frontal Gyrus; MOG, Middle Occipital Gyrus; PC, Precuneus; PCC, Posterior Cingulate Cortex; PHG, Parahippocampal Gyrus; R, Right; RC, Restrosplenial Cortex; SFG, Superior Frontal Gyrus.

t values are presented.

*p<0.05, **p<0.01.

**Table S19. The relationship between gray matter volume and functional connectivity in anterior salience network among AUD group.**

| **GMV**  **FC** | **L. MFG (1)** | **L. INS (2)** | ***ACC, SMA, MPFC (3)*** | **R. MFG (4)** | **R. INS (5)** | **L. LVI, CI (6)** | **R. LVI, CI (7)** |
| --- | --- | --- | --- | --- | --- | --- | --- |
| **1 – 2^a^** | -0.32 | -0.04 |  |  |  |  |  |
| **1 - 3^a, b^** | **-0.45^*^** |  | -0.06 |  |  |  |  |
| **1 - 4^a^** | 0.03 |  |  | -0.31 |  |  |  |
| **1 - 5^a^** | -0.26 |  |  |  | -0.02 |  |  |
| **1 - 6^a^** | 0.11 |  |  |  |  | -0.33 |  |
| **1 - 7^a^** | -0.07 |  |  |  |  |  | -0.24 |
| **2 - 3^a^** |  | -0.22 | **-0.48^*^** |  |  |  |  |
| **2 - 4^a^** |  | -0.22 |  | -0.27 |  |  |  |
| **2 - 5^a^** |  | -0.04 |  |  | 0.01 |  |  |
| **2 - 6^a^** |  | -0.15 |  |  |  | -0.20 |  |
| **2 - 7^a^** |  | -0.16 |  |  |  |  | -0.04 |
| **3 - 4^a^** |  |  | -0.15 | -0.34 |  |  |  |
| **3 - 5^a^** |  |  | 0.10 |  | -0.06 |  |  |
| **3 - 6^a^** |  |  | 0.05 |  |  | 0.09 |  |
| **3 - 7^a^** |  |  | 0.00 |  |  |  | -0.03 |
| **4 - 5^a^** |  |  |  | -0.11 | -0.09 |  |  |
| **4 - 6^a^** |  |  |  | -0.25 |  | -0.22 |  |
| **4 - 7^a^** |  |  |  | -0.01 |  |  | 0.13 |
| **5 - 6^a^** |  |  |  |  | 0.03 | -0.16 |  |
| **5 – 7** |  |  |  |  | 0.31 |  | -0.01 |
| **6 - 7^a^** |  |  |  |  |  | **0.52^*^** | 0.34 |

Abbreviations: ACC, Anterior Cingulate Cortex; AUD, Alcohol Use Disorder group; HC, Healthy Control group; INS, Insula; CI, Crus I; L, Left; LVI, Lobule VI; MFG, Middle Frontal Gyrus; MPFC, Medial Prefrontal Cortex; R, Right; SMA, Supplementary Motor Area.

The italicized regions showed the reductions of gray matter volume among AUD group.

^a^The variables showed the significant correlation of functional connectivity among AUD group.

^b^The variables showed the significant difference of functional connectivity between AUD and HC groups.

*p < 0.05, **p < 0.01, and ***p < 0.001.

**Table S20. The relationship between gray matter volume and functional connectivity in posterior salience network among AUD group.**

| **GMV**  **FC** | **L. MFG (1)** | **L. SMG, IPG (2)** | **L. PC (3)** | **R. MCC (4)** | **R. SPG, PC (5)** | **R. SMG, IPG (6)** | ***L. Th (7)*** | **R. LVI (8)** | **L. PI, Pu (9)** | **R. Th (10)** | ***L. LVI (11)*** | ***R.***  ***PI (12)*** |
| --- | --- | --- | --- | --- | --- | --- | --- | --- | --- | --- | --- | --- |
| **1 - 2^a^** | 0.14 | 0.17 |  |  |  |  |  |  |  |  |  |  |
| **1 – 3** | 0.22 |  | -0.01 |  |  |  |  |  |  |  |  |  |
| **1 – 4** | 0.21 |  |  | -0.08 |  |  |  |  |  |  |  |  |
| **1 – 5** | 0.18 |  |  |  | -0.05 |  |  |  |  |  |  |  |
| **1 - 6^a^** | 0.24 |  |  |  |  | **0.43^*^** |  |  |  |  |  |  |
| **1 – 7** | 0.26 |  |  |  |  |  | 0.11 |  |  |  |  |  |
| **1 – 8** | 0.16 |  |  |  |  |  |  | -0.02 |  |  |  |  |
| **1 – 9** | 0.24 |  |  |  |  |  |  |  | 0.16 |  |  |  |
| **1 – 10** | 0.11 |  |  |  |  |  |  |  |  | 0.11 |  |  |
| **1 – 11** | 0.22 |  |  |  |  |  |  |  |  |  | 0.05 |  |
| **1 – 12** | 0.15 |  |  |  |  |  |  |  |  |  |  | 0.02 |
| **2 - 3^a^** |  | -0.07 | 0.05 |  |  |  |  |  |  |  |  |  |
| **2 - 4^a^** |  | -0.23 |  | 0.09 |  |  |  |  |  |  |  |  |
| **2 - 5^a^** |  | -0.16 |  |  | 0.06 |  |  |  |  |  |  |  |
| **2 - 6^a^** |  | -0.02 |  |  |  | 0.31 |  |  |  |  |  |  |
| **2 – 7** |  | 0.23 |  |  |  |  | 0.07 |  |  |  |  |  |
| **2 - 8^a^** |  | 0.07 |  |  |  |  |  | 0.00 |  |  |  |  |
| **2 - 9^a^** |  | 0.12 |  |  |  |  |  |  | 0.12 |  |  |  |
| **2 – 10** |  | -0.02 |  |  |  |  |  |  |  | 0.17 |  |  |
| **2 – 11** |  | -0.32 |  |  |  |  |  |  |  |  | 0.07 |  |
| **2 - 12^a^** |  | 0.19 |  |  |  |  |  |  |  |  |  | -0.03 |
| **3 - 4^a^** |  |  | -0.30 | -0.09 |  |  |  |  |  |  |  |  |
| **3 - 5^a^** |  |  | -0.20 |  | 0.04 |  |  |  |  |  |  |  |
| **3 - 6^a^** |  |  | 0.04 |  |  | 0.13 |  |  |  |  |  |  |
| **3 – 7** |  |  | -0.13 |  |  |  | 0.14 |  |  |  |  |  |
| **3 – 8^a^** |  |  | 0.36 |  |  |  |  | 0.23 |  |  |  |  |
| **3 – 9^a^** |  |  | -0.04 |  |  |  |  |  | 0.05 |  |  |  |
| **3 – 10** |  |  | 0.10 |  |  |  |  |  |  | -0.04 |  |  |
| **3 – 11** |  |  | 0.38 |  |  |  |  |  |  |  | 0.33 |  |
| **3 - 12^a^** |  |  | **-0.49^*^** |  |  |  |  |  |  |  |  | -0.19 |
| **4 – 5^a^** |  |  |  | 0.08 | 0.22 |  |  |  |  |  |  |  |
| **4 – 6^a^** |  |  |  | 0.00 |  | -0.22 |  |  |  |  |  |  |
| **4 - 7^a^** |  |  |  | -0.37 |  |  | 0.04 |  |  |  |  |  |
| **4 – 8** |  |  |  | -0.13 |  |  |  | **0.43^*^** |  |  |  |  |
| **4 – 9^a^** |  |  |  | -0.11 |  |  |  |  | 0.18 |  |  |  |
| **4 – 10** |  |  |  | -0.45^*^ |  |  |  |  |  | 0.02 |  |  |
| **4 – 11** |  |  |  | 0.01 |  |  |  |  |  |  | 0.33 |  |
| **4 – 12^a^** |  |  |  | 0.04 |  |  |  |  |  |  |  | 0.32 |
| **5 – 6^a^** |  |  |  |  | 0.04 | -0.01 |  |  |  |  |  |  |
| **5 – 7** |  |  |  |  | -0.30 |  | -0.03 |  |  |  |  |  |
| **5 - 8^a^** |  |  |  |  | 0.03 |  |  | 0.09 |  |  |  |  |
| **5 - 9^a^** |  |  |  |  | -0.11 |  |  |  | 0.18 |  |  |  |
| **5 – 10** |  |  |  |  | -0.10 |  |  |  |  | -0.22 |  |  |
| **5 – 11** |  |  |  |  | 0.20 |  |  |  |  |  | 0.24 |  |
| **5 - 12^a^** |  |  |  |  | -0.01 |  |  |  |  |  |  | -0.02 |
| **6 – 7** |  |  |  |  |  | 0.13 | 0.13 |  |  |  |  |  |
| **6 - 8^a^** |  |  |  |  |  | -0.04 |  | 0.16 |  |  |  |  |
| **6 - 9^a^** |  |  |  |  |  | 0.05 |  |  | 0.14 |  |  |  |
| **6 – 10^b^** |  |  |  |  |  | -0.01 |  |  |  | 0.15 |  |  |
| **6 – 11** |  |  |  |  |  | -0.37 |  |  |  |  | 0.02 |  |
| **6 - 12^a^** |  |  |  |  |  | 0.13 |  |  |  |  |  | 0.12 |
| **7 – 8** |  |  |  |  |  |  | -0.05 | **0.46^*^** |  |  |  |  |
| **7 – 9** |  |  |  |  |  |  | 0.37 |  | 0.12 |  |  |  |
| **7 - 10^a^** |  |  |  |  |  |  | 0.23 |  |  | 0.33 |  |  |
| **7 – 11** |  |  |  |  |  |  | -0.01 |  |  |  | 0.32 |  |
| **7 – 12** |  |  |  |  |  |  | 0.60^**^ |  |  |  |  | -0.05 |
| **8 – 9** |  |  |  |  |  |  |  | 0.19 | 0.12 |  |  |  |
| **8 – 10** |  |  |  |  |  |  |  | 0.28 |  | 0.17 |  |  |
| **8 - 11^a^** |  |  |  |  |  |  |  | -0.09 |  |  | 0.11 |  |
| **8 – 12^a^** |  |  |  |  |  |  |  | 0.18 |  |  |  | 0.08 |
| **9 – 10** |  |  |  |  |  |  |  |  | 0.05 | 0.23 |  |  |
| **9 – 11** |  |  |  |  |  |  |  |  | -0.29 |  | -0.02 |  |
| **9 - 12^a^** |  |  |  |  |  |  |  |  | **0.43^*^** |  |  | 0.37 |
| **10 – 11** |  |  |  |  |  |  |  |  |  | 0.19 | 0.10 |  |
| **10 – 12** |  |  |  |  |  |  |  |  |  | 0.37 |  | **-0.43^*^** |
| **11 – 12** |  |  |  |  |  |  |  |  |  |  | 0.16 | 0.20 |

Abbreviations: AUD, Alcohol Use Disorder group; HC, Healthy Control group; IPG, Inferior Parietal Gyrus; L, Left; LVI, Lobule VI; MCC, Midcingulate cortex; MFG, Middle Frontal Gyrus; PC, Precuneus; R, Right; PI Posterior Insula; Pu, Putamen; SMG, Supramarginal gyrus; SPG, Superior Parietal Gyrus; Th, Thalamus.

The italicized regions showed the reductions of gray matter volume among AUD group.

^a^The variables showed the significant correlation of functional connectivity among AUD group.

^b^The variables showed the significant difference of functional connectivity between AUD and HC groups.

*p < 0.05, **p < 0.01, and ***p < 0.001.

**Table S21. The relationship between gray matter volume and functional connectivity in left executive control network among AUD group.**

| **GMV**  **FC** | ***L. MFG, SFG (1)*** | ***L. IFG, OFG (2)*** | **L. SPG, IPG, PC, AG (3)** | **L. ITG, MTG (4)** | **R. CI (5)** | **L. Th (6)** |
| --- | --- | --- | --- | --- | --- | --- |
| **1 – 2^a^** | -0.22 | -0.08 |  |  |  |  |
| **1 - 3^a^** | 0.03 |  | -0.06 |  |  |  |
| **1 - 4^a^** | -0.03 |  |  | -0.28 |  |  |
| **1 - 5^a^** | -0.13 |  |  |  | 0.25 |  |
| **1 – 6** | 0.28 |  |  |  |  | -0.15 |
| **2 - 3^a^** |  | 0.15 | -0.20 |  |  |  |
| **2 - 4^a^** |  | -0.11 |  | -0.12 |  |  |
| **2 - 5^a^** |  | 0.04 |  |  | 0.31 |  |
| **2 – 6** |  | -0.02 |  |  |  | -0.37 |
| **3 - 4^a^** |  |  | 0.38 | -0.25 |  |  |
| **3 - 5^a^** |  |  | 0.21 |  | 0.20 |  |
| **3 – 6** |  |  | -0.13 |  |  | -0.29 |
| **4 - 5^a^** |  |  |  | -0.25 | 0.06 |  |
| **4 - 6** |  |  |  | -0.09 |  | -0.26 |
| **5 - 6** |  |  |  |  | 0.31 | -0.26 |

Abbreviations: AG, Angular gyrus; AUD, Alcohol Use Disorder group; HC, Healthy Control group; CI, Crus I; IFG, Inferior frontal gyrus; ITG, Inferior temporal gyrus; L, Left; MFG, Middle Frontal Gyrus; MTG, middle temporal gyrus; PC, precuneus; R, Right; SFG, Superior frontal cortex; SPG, superior parietal gyrus.

The italicized regions showed the reductions of gray matter volume among AUD group.

^a^The variables showed the significant correlation of functional connectivity among AUD group.

^b^The variables showed the significant difference of functional connectivity between AUD and HC groups.

*p < 0.05, **p < 0.01, and ***p < 0.001.

**Table S22. The relationship between gray matter volume and functional connectivity in right executive control network among AUD group.**

| **GMV**  **FC** | **R. MFG, SFG (1)** | **R. MFG (2)** | **R. IPG, SMG, AG (3)** | **R. SFG (4)** | ***L. LVI, CI, CII (5)*** | **R. Cau (6)** |
| --- | --- | --- | --- | --- | --- | --- |
| **1 - 2^a^** | 0.35 | 0.26 |  |  |  |  |
| **1 - 3^a, b^** | 0.14 |  | 0.00 |  |  |  |
| **1 - 4^a^** | -0.15 |  |  | 0.33 |  |  |
| **1 - 5^a^** | -0.37 |  |  |  | -0.15 |  |
| **1 - 6^a^** | -0.28 |  |  |  |  | 0.11 |
| **2 - 3^a^** |  | 0.32 | 0.20 |  |  |  |
| **2 - 4^a^** |  | 0.04 |  | 0.07 |  |  |
| **2 - 5^a^** |  | 0.06 |  |  | 0.19 |  |
| **2 – 6** |  | 0.15 |  |  |  | 0.01 |
| **3 - 4^a^** |  |  | -0.04 | **0.43^*^** |  |  |
| **3 - 5^a^** |  |  | -0.38 |  | -0.18 |  |
| **3 - 6^a^** |  |  | -0.19 |  |  | 0.16 |
| **4 - 5^a^** |  |  |  | -0.09 | -0.31 |  |
| **4 - 6^a^** |  |  |  | 0.34 |  | 0.29 |
| **5 - 6^a^** |  |  |  |  | 0.07 | 0.09 |

Abbreviations: AG, Angular gyrus; AUD, Alcohol Use Disorder group; HC, Healthy Control group; CI, Crus I; CII, Crus II; L, Left; LVI, Lobule VI; MFG, Middle Frontal Gyrus; R, Right; SFG, Superior frontal gyrus; SMG, Supramarginal gyrus.

The italicized regions showed the reductions of gray matter volume among AUD group.

^a^The variables showed the significant correlation of functional connectivity among AUD group.

^b^The variables showed the significant difference of functional connectivity between AUD and HC groups.

*p < 0.05, **p < 0.01, and ***p < 0.001.

**Table S23. The relationship between gray matter volume and functional connectivity in dorsal default mode network among AUD group.**

| **GMV**  **FC** | ***MPFC, ACC, OFC (1)*** | **L. AG (2)** | **R. SFG (3)** | **PCC, PC (4)** | **MCC (5)** | **R. AG (6)** | **Th (7)** | ***L. Hip (8)*** | ***R. Hip (9)*** |
| --- | --- | --- | --- | --- | --- | --- | --- | --- | --- |
| **1 – 2^a^** | 0.06 | 0.14 |  |  |  |  |  |  |  |
| **1 - 3^a^** | 0.08 |  | -0.05 |  |  |  |  |  |  |
| **1 - 4^a^** | -0.02 |  |  | 0.27 |  |  |  |  |  |
| **1 - 5^a^** | 0.13 |  |  |  | 0.03 |  |  |  |  |
| **1 – 6^a^** | -0.09 |  |  |  |  | 0.17 |  |  |  |
| **1 – 7^a^** | 0.13 |  |  |  |  |  | -0.02 |  |  |
| **1 - 8^a^** | -0.03 |  |  |  |  |  |  | 0.06 |  |
| **1 - 9^a^** | -0.19 |  |  |  |  |  |  |  | -0.12 |
| **2 - 3^a^** |  | 0.19 | 0.02 |  |  |  |  |  |  |
| **2 - 4^a^** |  | 0.20 |  | 0.21 |  |  |  |  |  |
| **2 - 5^a^** |  | 0.31 |  |  | -0.29 |  |  |  |  |
| **2 - 6^a^** |  | -0.30 |  |  |  | -0.03 |  |  |  |
| **2 – 7** |  | 0.09 |  |  |  |  | 0.12 |  |  |
| **2 - 8^a^** |  | 0.11 |  |  |  |  |  | -0.02 |  |
| **2 - 9^a^** |  | 0.01 |  |  |  |  |  |  | -0.10 |
| **3 - 4^a^** |  |  | 0.06 | -0.29 |  |  |  |  |  |
| **3 - 5^a^** |  |  | 0.18 |  | 0.26 |  |  |  |  |
| **3 - 6^a^** |  |  | 0.05 |  |  | 0.12 |  |  |  |
| **3 – 7** |  |  | 0.35 |  |  |  | **0.45^*^** |  |  |
| **3 - 8^a^** |  |  | 0.16 |  |  |  |  | -0.06 |  |
| **3 - 9^a^** |  |  | 0.12 |  |  |  |  |  | 0.02 |
| **4 - 5^a^** |  |  |  | **0.42^*^** | -0.04 |  |  |  |  |
| **4 - 6^a^** |  |  |  | 0.21 |  | 0.20 |  |  |  |
| **4 - 7^a^** |  |  |  | 0.03 |  |  | 0.04 |  |  |
| **4 - 8^a^** |  |  |  | 0.10 |  |  |  | -0.23 |  |
| **4 - 9^a^** |  |  |  | 0.03 |  |  |  |  | -0.32 |
| **5 - 6^a^** |  |  |  |  | -0.04 | **0.44^*^** |  |  |  |
| **5 - 7^a^** |  |  |  |  | 0.36 |  | 0.39 |  |  |
| **5 - 8^a^** |  |  |  |  | 0.32 |  |  | 0.27 |  |
| **5 - 9^a^** |  |  |  |  | 0.17 |  |  |  | 0.11 |
| **6 – 7** |  |  |  |  |  | 0.24 | 0.19 |  |  |
| **6 - 8^a, b^** |  |  |  |  |  | 0.02 |  | **-0.42^*^** |  |
| **6 - 9^a, b^** |  |  |  |  |  | -0.20 |  |  | **-0.55^**^** |
| **7 - 8^a^** |  |  |  |  |  |  | -0.10 | -0.07 |  |
| **7 – 9** |  |  |  |  |  |  | -0.16 |  | 0.19 |
| **8 - 9^a, b^** |  |  |  |  |  |  |  | 0.35 | **-0.45^*^** |

Abbreviations: ACC, Anterior Cingulate Cortex; AG, Angular Gyrus; AUD, Alcohol Use Disorder group; HC, Healthy Control group; Hip, Hippocampus; L, Left; MCC, Midcingulate Cortex; MPFC, Medial Prefrontal Cortex; OFC, Orbitofrontal cortex; PC, Precuneus; PCC, Posterior Cingulate Cortex; R, Right; SFG, Superior Frontal gyrus; Th, Thalamus.

The italicized regions showed the reductions of gray matter volume among AUD group.

^a^The variables showed the significant correlation of functional connectivity among AUD group.

^b^The variables showed the significant difference of functional connectivity between AUD and HC groups.

*p < 0.05, **p < 0.01, and ***p < 0.001.

**Table S24. The relationship between gray matter volume and functional connectivity in ventral default mode network among AUD group.**

| **GMV**  **FC** | **L. RC, PCC (1)** | **L. MFG**  **(2)** | ***L. PHG***  ***(3)*** | **L. MOG**  **(4)** | **R. RC, PCC (5)** | **PC**  **(6)** | **R. SFG, MFG (7)** | ***R. PHG***  ***(8)*** | **R. AG, MOG (9)** | ***R. L IX***  ***(10)*** |
| --- | --- | --- | --- | --- | --- | --- | --- | --- | --- | --- |
| **1 – 2** | 0.36 | 0.03 |  |  |  |  |  |  |  |  |
| **1 - 3^a^** | -0.23 |  | -0.17 |  |  |  |  |  |  |  |
| **1 - 4^a^** | 0.30 |  |  | -0.20 |  |  |  |  |  |  |
| **1 – 5^a^** | 0.04 |  |  |  | 0.07 |  |  |  |  |  |
| **1 - 6^a^** | 0.01 |  |  |  |  | -0.04 |  |  |  |  |
| **1 - 7^a^** | 0.31 |  |  |  |  |  | 0.14 |  |  |  |
| **1 - 8^a^** | 0.00 |  |  |  |  |  |  | **-0.50^*^** |  |  |
| **1 - 9^a^** | 0.13 |  |  |  |  |  |  |  | 0.18 |  |
| **1 - 10** | 0.06 |  |  |  |  |  |  |  |  | 0.35 |
| **2 - 3^a^** |  | -0.11 | -0.26 |  |  |  |  |  |  |  |
| **2 - 4^a^** |  | -0.30 |  | -0.41 |  |  |  |  |  |  |
| **2 – 5** |  | -0.04 |  |  | 0.26 |  |  |  |  |  |
| **2 - 6^a^** |  | 0.36 |  |  |  | 0.24 |  |  |  |  |
| **2 - 7^a^** |  | -0.30 |  |  |  |  | -0.09 |  |  |  |
| **2 - 8^a^** |  | 0.12 |  |  |  |  |  | -0.32 |  |  |
| **2 - 9^a, b^** |  | -0.19 |  |  |  |  |  |  | 0.05 |  |
| **2 – 10** |  | 0.27 |  |  |  |  |  |  |  | 0.35 |
| **3 - 4^a^** |  |  | 0.09 | **-0.51^*^** |  |  |  |  |  |  |
| **3 - 5^a^** |  |  | -0.15 |  | -0.12 |  |  |  |  |  |
| **3 - 6^a^** |  |  | -0.06 |  |  | -0.15 |  |  |  |  |
| **3 - 7^a^** |  |  | -0.40 |  |  |  | -0.11 |  |  |  |
| **3 - 8^a^** |  |  | 0.17 |  |  |  |  | -0.10 |  |  |
| **3 - 9^a^** |  |  | 0.10 |  |  |  |  |  | 0.19 |  |
| **3 – 10** |  |  | -0.24 |  |  |  |  |  |  | 0.29 |
| **4 - 5^a^** |  |  |  | -0.26 | 0.25 |  |  |  |  |  |
| **4 - 6^a^** |  |  |  | -0.40 |  | -0.19 |  |  |  |  |
| **4 - 7^a^** |  |  |  | -0.31 |  |  | -0.10 |  |  |  |
| **4 - 8^a^** |  |  |  | -0.32 |  |  |  | -0.10 |  |  |
| **4 - 9^a^** |  |  |  | 0.11 |  |  |  |  | 0.06 |  |
| **4 – 10** |  |  |  | 0.07 |  |  |  |  |  | 0.22 |
| **5 - 6^a^** |  |  |  |  | -0.18 | -0.09 |  |  |  |  |
| **5 - 7^a^** |  |  |  |  | 0.29 |  | 0.11 |  |  |  |
| **5 - 8^a^** |  |  |  |  | 0.07 |  |  | **-0.44^*^** |  |  |
| **5 - 9^a^** |  |  |  |  | 0.27 |  |  |  | 0.20 |  |
| **5 – 10** |  |  |  |  | 0.00 |  |  |  |  | 0.39 |
| **6 - 7^a^** |  |  |  |  |  | -0.06 | 0.02 |  |  |  |
| **6 - 8^a^** |  |  |  |  |  | 0.01 |  | -0.24 |  |  |
| **6 - 9^a^** |  |  |  |  |  | -0.34 |  |  | -0.23 |  |
| **6 - 10^a, b^** |  |  |  |  |  | 0.39 |  |  |  | 0.40 |
| **7 - 8^a, b^** |  |  |  |  |  |  | -0.02 | **-0.60^**^** |  |  |
| **7 - 9^a^** |  |  |  |  |  |  | -0.01 |  | 0.37 |  |
| **7 - 10** |  |  |  |  |  |  | 0.10 |  |  | 0.23 |
| **8 - 9^a, b^** |  |  |  |  |  |  |  | -0.39 | -0.05 |  |
| **8 – 10** |  |  |  |  |  |  |  | **-0.51^*^** |  | 0.21 |
| **9 – 10** |  |  |  |  |  |  |  |  | -0.18 | -0.03 |

Abbreviations: AG, Angular Gyrus; AUD, Alcohol Use Disorder group; HC, Healthy Control group; L, Left; LIX, Lobule IX; MFG, Middle Frontal Gyrus; MOG, Middle Occipital Gyrus; PC, Precuneus; PCC, Posterior Cingulate Cortex; PHG, Parahippocampal Gyrus; R, Right; RC, Restrosplenial Cortex; SFG, Superior Frontal Gyrus.

The italicized regions showed the reductions of gray matter volume among AUD group.

^a^The variables showed the significant correlation of functional connectivity among AUD group.

^b^The variables showed the significant difference of functional connectivity between AUD and HC groups.

*p < 0.05, **p < 0.01, and ***p < 0.001.
